# Role of Antioxidant Therapy in the Treatment and Prognosis of COVID-19: A Systematic Review and Meta-analysis of Randomized Controlled Trials

**DOI:** 10.1016/j.cdnut.2024.102145

**Published:** 2024-03-24

**Authors:** Radha Sharma, Atushi Patel, Tanvi Ojha, Lesley A Pablo, Tina Vosoughi, Carolyn Ziegler, Krishihan Sivapragasam, Andrew D Pinto, David Jenkins, Banafshe Hosseini

**Affiliations:** 1 Upstream Lab, MAP Centre for Urban Health Solutions, Li Ka Shing Knowledge Institute, St. Michael’s Hospital, Toronto, Ontario, Canada; 2 Temerty Faculty of Medicine, University of Toronto, Toronto, Ontario, Canada; 3 Library Services, Unity Health Toronto, Toronto, Ontario, Canada; 4 Department of Family and Community Medicine, St. Michael’s Hospital, Toronto, Ontario, Canada; 5 Department of Family and Community Medicine, Faculty of Medicine, University of Toronto, Toronto, Ontario, Canada; 6 Division of Clinical Public Health & Institute for Health Policy, Management and Evaluation, Dalla Lana School of Public Health, University of Toronto, Toronto, Ontario, Canada; 7 Department of Nutritional Sciences, Temerty Faculty of Medicine, University of Toronto, Toronto, Ontario, Canada; 8 Division of Endocrinology and Metabolism, St. Michael’s Hospital, Toronto, Ontario, Canada

**Keywords:** COVID-19, SARS-CoV-2, antioxidant therapy, randomized controlled trials, systematic review

## Abstract

**Background:**

A significant aspect of the SARS-CoV-2 pathology involves oxidative stress, characterized by an imbalance between the production of harmful free radicals and the body’s antioxidant defenses. With the ongoing evolution of SARS-CoV-2, the investigation into non–virus-specific therapeutic options, such as antioxidant therapy, has gained importance.

**Objectives:**

This systematic review and meta-analysis aimed to summarize data from randomized control trials (RCTs) to evaluate the effectiveness and safety of antioxidant therapy in patients with SARS-CoV-2 infection.

**Methods:**

We searched the peer-reviewed indexed literature on MEDLINE, Cochrane Central Register of Controlled Trials (CENTRAL), CINAHL, EMBASE, International Pharmaceutical Abstracts, and Scopus, from inception to July 2023.

**Results:**

The search identified 3306 articles from which 25 were included for quantitative synthesis, with 5 studies eligible for meta-analysis. Antioxidant therapies included zinc, vitamin A, vitamin C, and combination treatments. Zinc interventions showed mixed results regarding intensive care unit admissions and hospital stays. Vitamin A studies indicated improvements in inflammatory markers. Vitamin C studies displayed inconsistent effects on clinical improvement and hospitalization. Combination treatments suggested benefits in symptom clearance and cytokine storm reduction. Meta-analysis of vitamin C studies found no significant difference in C-reactive protein concentrations (−0.50; 95% CI: −3.63, 2.63; *I*^2^ = 0%), intensive care unit stay duration (pooled mean difference: 1.44; 95% CI: 0.07, 2.81; *I*^2^ = 0%), or mortality (pooled odds ratio: 0.55; 95% CI: 0.28, 1.09; *I*^2^ = 0%), with a slight trend favoring reduced hospitalization duration (pooled mean difference: −2.37; 95% CI: −2.99, −1.76; *I*^2^ = 49%). Of the 25 studies, 8 were high quality with low bias, 6 had some concerns, and 11 were low quality with high bias.

**Conclusions:**

The review presents mixed efficacy of antioxidant therapies for SARS-CoV-2, with some studies indicating potential benefits. Further well-designed large-scale RCTs are warranted to determine the definitive role of antioxidants in SARS-CoV-2 treatment.

This systematic review was registered at PROSPERO as CRD42023430805.

## Introduction

As SARS-CoV-2 transitions from a pandemic status toward potential endemicity, the need for effective treatments that are not specific to a particular strain becomes even more pronounced [1]. The overwhelming of acute health service bed capacity presents a significant challenge to health care systems. Reducing inpatient hospital stays for patients with COVID has a profound impact on community health care access. Although various interventions have been explored, a definitive treatment protocol for SARS-CoV-2 remains elusive [1,2]. The depletion of antiviral defenses and elevated production of inflammatory cytokines have been identified as key factors in SARS-CoV-2 infection. The resulting cytokine storm contributes significantly to the pathophysiology of SARS-CoV-2, potentially leading to severe complications such as acute respiratory distress syndrome, multiple organ failure, and mortality [3].

Antioxidant therapy, which reduces the pro-oxidant tissue damage associated with the response to viral infections, has gained attention for its potential in managing SARS-CoV-2 [4]. This therapeutic approach, involving vitamins C and E, selenium, zinc, and other antioxidants, can restore the balance between pro-oxidant and antioxidant mechanisms and modulate oxidative stress and inflammation, thereby mitigating the severity of the cytokine storm and its associated complications [5]. Emerging research provides partial validation for these hypotheses on antioxidant therapy for SARS-CoV-2. A systematic review of 11 studies found mostly negative associations between disease severity and serum selenium concentrations, with increased renal excretion of selenium correlating with disease severity in 1 study [6]. Likewise, trials have demonstrated the potential benefits of selenium supplementation in improving immune response [7,8]. Moreover, zinc supplementation, as documented in a Cochrane review, showed reduced common cold symptoms and antibiotic use [9]. Furthermore, a meta-analysis reported lower risk of mortality associated with zinc supplementation [10].

Despite these findings, evidence on the effectiveness and safety of antioxidant therapy in SARS-CoV-2 remain inconclusive. Conflicting results persist regarding mortality rates, hospital stay duration, and intensive care unit (ICU) admission requirements [11]. This proposed systematic review and meta-analysis aimed to summarize data from randomized controlled trials (RCTs) to draw robust and reliable conclusions on the effectiveness and safety of antioxidant therapy in patients with SARS-CoV-2 infection. This review intended to provide updated evidence to inform clinical guidelines and direct future research efforts, offering a clearer picture of the role of antioxidants in managing this formidable disease.

## Methods

This systematic review followed the Preferred Reporting Items for Systematic Reviews and Meta-Analyses Extension for Systematic Reviews (PRISMA) guidelines [12]. We registered this systematic review with PROSPERO (registration number: CRD42023430805).

### Databases

A systematic search was conducted by an Information Specialist from the Unity Health Library (CZ), on July 6, 2023, in MEDLINE (Ovid), Cochrane Central Register of Controlled Trials (CENTRAL) (Ovid), CINAHL (EBSCOhost), EMBASE (Ovid), International Pharmaceutical Abstracts (Ovid), and Scopus. The databases were searched from inception to July 6, 2023 (inclusive).

### Search strategy

The search strategies used a comprehensive combination of medical subject headings and keywords for the following concepts: (COVID-19 OR SARS-CoV-2 OR coronavirus OR Coronaviridae OR Severe Acute Respiratory Distress Syndrome OR Severe acute respiratory syndrome coronavirus) AND (antioxidant OR vitamin A OR Ascorbic Acid OR vitamin E OR selenium OR zinc OR copper OR carotenoids OR lycopene) AND Randomized Controlled Trials. All search strategies as analyzed can be found in Supplemental Methods 1.

### Eligibility criteria

Studies were eligible if they were RCTs and tested the impact of one or more of the following antioxidant micronutrients: vitamin A, retinol, ascorbic acid, vitamin C, vitamin E, tocopherols, carotenes, lycopene, selenium, zinc, and copper against placebo or no treatment or standard of care in acute cases of SARS-CoV-2. The following designs were excluded: animal models, quasiexperimental, cross-sectional studies, in vitro studies, systematic reviews, narrative reviews, opinion articles, case studies, and conference articles. Studies were also excluded if testing nonantioxidant interventions. Non-English studies were also removed owing to lack of robust resources to accurately translate material.

Study participants were humans of all ages, sexes, or ethnicities who were infected with SARS-CoV-2. The intervention was antioxidant supplementation provided in any dose, format (oral compared with nonoral), or frequency. Subjects who received antioxidant supplementation comprised the intervention arm, whereas those who received placebo or no antioxidant supplementation constituted the control arm. Standard therapy was permitted as a cointervention, if provided to both groups.

The primary outcomes of interest included time to recovery, all-cause and COVID-related mortality, duration of ICU and hospital stays, necessity for artificial ventilation, changes in inflammatory biomarkers [such as C-reactive protein (CRP), interleukin (IL)-6, and D-dimer], severity and duration of symptoms, and presence of long COVID.

### Screening process

EndNote software X9.2 was used to compile the search results and deduplicate the records. Covidence was used throughout the review to manage citations. Trained individuals (AP, RS, TO, TV) screened citations using the eligibility criteria to determine the inclusion or exclusion of studies. First-level screening consisted of title and abstract screening of all uploaded studies. Each citation was reviewed by 2 people independently to select studies for full-text review (RS, TO). If the eligibility criteria were met completely, as assessed by both reviewers, the studies were included. If studies did not meet eligibility criteria, as determined by both reviewers, they were excluded. Any citations where there was a difference in opinion were brought to the study team to discuss and a third reviewer decided on inclusion or exclusion (AP or TV). Second-level screening involved an assessment of the full-text of all studies that passed the title and abstract screening, performed by a solo reviewer (AP, RS, or TO), who excluded any studies that did not meet the same eligibility criteria. The final set of studies included in this systematic review includes only those reports that passed full-text screening.

### Data collection and synthesis

Two members of the study team independently performed data extraction from all studies (RS, TO). To extract data from the included studies, an extraction form was uploaded onto Covidence (Supplemental Methods 2), which was developed using the Cochrane guidelines [13]. Pilot testing with the form was completed on 5 randomly selected studies by 2 reviewers (RS, TO). The data extraction was then checked for consensus by 1 member of the study team (RS).

Data were collected on the meta-data (author name, year of publication, and journal), antioxidant type(s) and method of delivery, whether treatment was in combination or on its own, the intended purpose of the study, study design, research question(s), results, population data including jurisdiction, unit(s) of analysis, sample size, and demographics. Data on bias, target population, and impact were also extracted. If information was not available from an article, it was noted.

Eligible studies were categorized based on similar intervention groups and presented in tabular format using data obtained from the extraction form. Summary statistics alongside pertinent summarized study information was presented in tables created in Microsoft Word. Narrative summaries were conducted on the extraction categories. Mean difference (MD), odds ratios (ORs), and hazard ratios (HRs) were extracted and included in the tables, where appropriate. For studies that reported medians and interquartile ranges, means and standard deviations (SDs) were estimated using the Box–Cox method [14]. For continuous outcomes with unreported effect measures, MD was calculated from the mean ± SD and number of participants. For dichotomous outcomes, with unreported effect measures, ORs or HRs were calculated from the number of participants in the intervention group and comparator groups.

### Risk of bias and quality assessment

Eligible studies were assessed independently for their methodologic quality by 2 reviewers (RS, TO). Methodologic quality was assessed by the Cochrane Handbook risk of bias tool (RoB-2) [15,16] based on the random sequence generation, allocation concealment, blinding of participants and personnel, blinding of outcome assessment, incomplete outcome data, selective reporting, and other sources of bias, including industry funding. Each study was rated as low risk of bias, some concerns, or high risk of bias. Any discordance on methodologic quality was solved by consensus or input of the third reviewer (KS). Findings were formatted and reported using robvis figures [15,17].

### Statistical methods

The meta-analysis was performed using Review Manager (RevMan, version 5.3; Nordic Cochrane Centre) [18]. Appreciable heterogeneity was assumed if *I*^2^ > 50% and *P* < 0.1. Meta-analysis was performed using fixed-effect modeling if *I*^2^ < 50% and random-effect modeling was used if *I*^2^ ≥ 50. In the case included studies used different interventions, a meta-analysis was performed in studies with similar outcome measurements. Forest plots, created in RevMan, presented combined effect measures for each meta-analysis conducted. Causes of heterogeneity were sought through subgroup analysis, when appropriate.

Sensitivity analysis was planned to be performed on primary outcomes, factoring out studies identified with an uncertain or high risk of bias to ensure validity and robustness of the findings. However, owing to the limited number of studies with low bias, this analysis was not completed in this review.

## Results

### Search results

**FIGURE 1:**
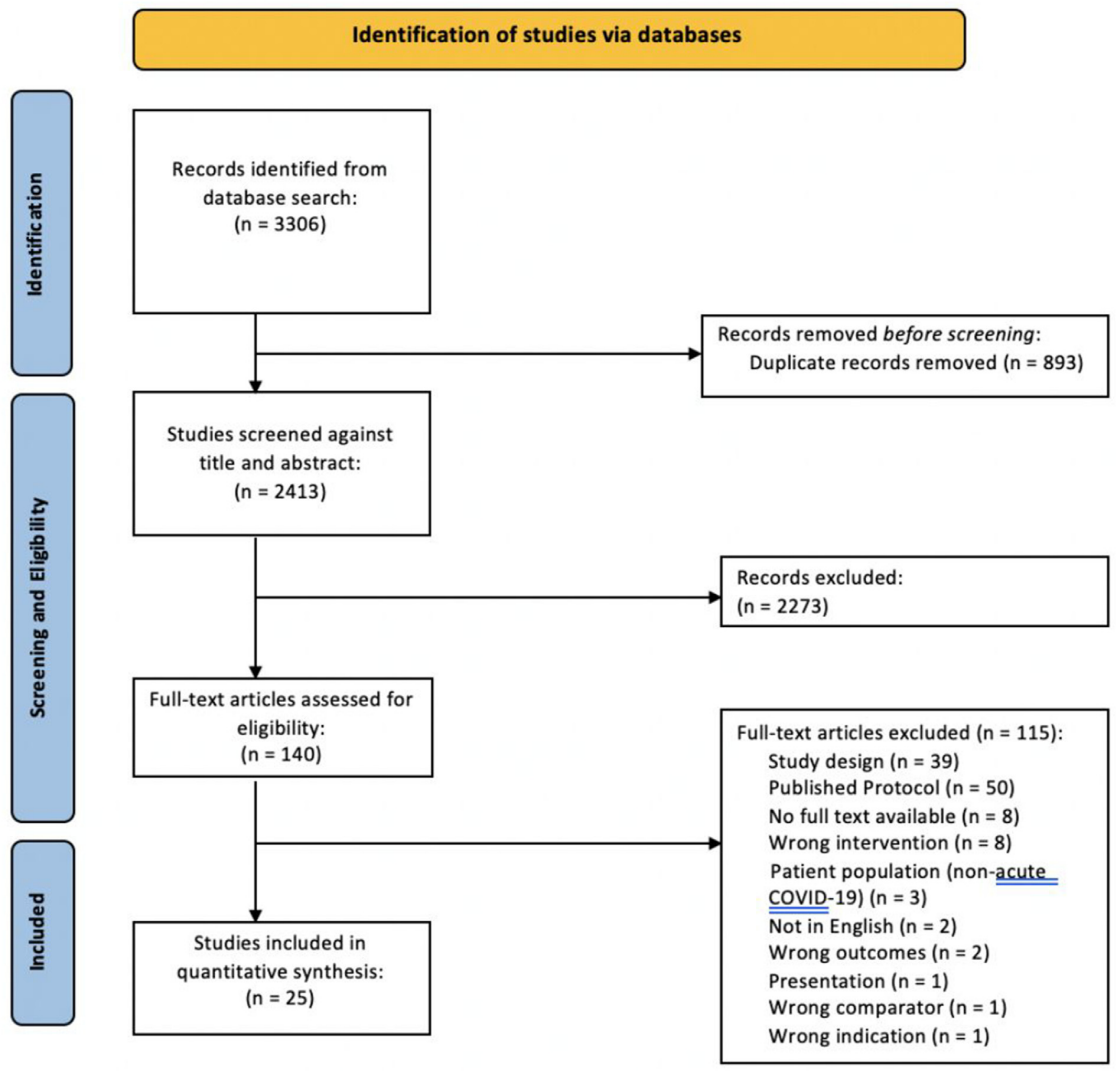
PRISMA flow diagram: selection process of eligible studies from all identified citations.

The initial search yielded 3306 total citations (Figure 1); 2413 results were identified following deduplication in EndNote X9.2. Screening was done independently by 4 reviewers (AP, RS, TO, TV). All included abstracts were assessed for inclusion or exclusion using the eligibility criteria, as discussed in the Methods section. In total, 140 abstracts were identified to enter second-level screening whereas 2273 records were excluded. During the full-text review, a further 115 were excluded following thorough assessment by 1 of 3 reviewers (AP, RS, TO). Ultimately, 25 articles were included in the quantitative synthesis included in this review.

An in-depth assessment and organization of the data (including outcomes, interventions, and population characteristics) was consequently completed to identify articles suitable for the meta-analysis. Based on this assessment, 5 studies were included in the meta-analysis.

### Study characteristics

Supplemental Table 1 presents the key data extracted from each included study. Studies included were published between 2020 and 2023, with 1 published in 2020 [19], 12 in 2021 [[7], [20], [21], [22], [23], [24], [25], [26], [27], [29], [30], [31]], 10 in 2022 [[32], [33], [34], [35], [36], [37], [38], [39], [40], [41]], and 2 in 2023 [42,43].

Geographic setting varied across the studies, with 9 studies from Iran [[20], [21], [22], [23],[32], [33], [34], [35], [36]], 3 from the United States [24,37,43], 2 from China [25,39], 2 from Tunisia [42,43], 2 from India [7,41] and the remaining 7, with 1 each from Egypt [26], Saudi Arabia [27], Israel [40], Pakistan [19], Australia [29], Spain [30], and Turkey [31].

The study populations were primarily in-patients with SARS-CoV-2 infection and moderate disease severity, as seen in 19 studies [[7], [19], [20], [21], [22], [23],[25], [26], [27],[29], [30], [31], [32], [33],^35^,^36^,[38], [39], [40]], whereas 6 focused on outpatients exhibiting acute symptoms [24,28,34,37,41,42]. Interestingly, 1 study assessed both inpatient and outpatient groups [43].

The main objective across these studies was to test the efficacy of antioxidant interventions on COVID-related outcomes. The major categories included zinc, vitamin A, vitamin C, and combination treatments. The search included selenium, vitamin E, copper, carotenoids, and lycopene, yet no studies were found.

The study sample size ranged from 20 to 470 participants across both intervention and comparator groups. Follow-up periods ranged from 7 to 30 d. Demographic data differed across studies, with mean age ranging from 31 to 70 y. All included studies were RCTs.

Regarding the specific interventions, 3 studies (12%) [26,29,43] assessed the impact of zinc interventions on outcomes such as time to recovery, mortality, number of ICU admissions, hospitalization length and rate, symptom duration, and requirement for oxygen support (Table 1). Two studies (8%) [34,35] assessed the impact of vitamin A on symptom duration, oxygen saturation, number and duration of hospitalizations, mortality, use of invasive mechanical ventilation, and change in inflammatory markers (Table 2). Vitamin C, either alone or in combination with standard of care treatment, was evaluated in 8 (32%) studies [19,22,23,25,33,[36], [37], [38]] (Table 3), with outcomes including clinical improvement, time to discharge, number and duration of hospitalizations, inflammatory marker changes, mortality, and the need for mechanical ventilation. Table 4 outlines the studies that used a combination of antioxidants, minerals, and/or multivitamins as their intervention [[7], [20],^21^,^24^,^27^,[30], [31], [32],[39], [40], [41], [42]]. Eleven (44%) of the articles reported outcomes such as clinical improvement, supplemental oxygen use, duration of ICU admission, inflammatory marker changes, and mortality. The effectiveness of these interventions was primarily defined by their statistical significance.

**TABLE 1:** Summary of studies—zinc interventions for patients with COVID-19

Study ID	Country	Setting	Outcomes	Treatment arm (*n*)	Control arm (*n*)	Statistic	Treatment vs. placebo	*P*	Mean difference (95% CI)	Odds ratio (95% CI)
Abdelmaksoud et al. [26]	Egypt	Inpatient	Time to recovery	Zinc, 50 mg twice daily until recovery (49)	Egyptian protocol treatment (56)	Median days (range)	12 (8–17) vs. 12 (8–20)	>0.05	7.25 (5.45, 9.05)	
Ben Abdallah et al. [43]	Tunisia	Inpatient/outpatient	Mortality	Oral zinc, 25 mg twice a day for 15 d (231) Inpatient (146)	Placebo capsule twice a day for 15 d (239) Inpatient (134)	Proportion (*n*)	15 vs.22	0.27		0.68 (0.34, 1.35)
			ICU admission			Proportion (*n*)	12 vs.27	0.01		0.43 (0.21, 0.87)
			Length of hospital stay (inpatient, *n* = 280)			Mean ± SD (d)	7.1 ± 3.4 vs. 10.6 ± 2.8	<0.001	3.5 (2.76, 4.23)	
			Duration of symptoms (outpatient, *n* = 190)			Mean ± SD (d)	9.6 ± 4.1 vs. 12.8 ± 6.7		1.9 (0.62, 2.6)	
			Hospital admission rate (outpatient, *n* = 190)			Admission rate (%)	1.20 vs. 3.80			0.30 (0.03, 2.8)
Patel et al. [29]	Australia	Inpatient	No. of nonventilated patients requiring oxygen support	Intravenous zinc, 0.24 mg/kg/d for maximum of 7 d (or until hospital discharge/death) (15)	Saline placebo for 7 d (18)	Proportion (*n*)	Day 1: 6 vs. 9 Day 7: 2 vs. 6	0.19		0.31 (0.05, 1.83)

**TABLE 2:** Summary of studies—vitamin A interventions

Study ID	Country	Setting	Outcomes	Treatment arm (*n*)	Control arm (*n*)	Statistic	Treatment vs. placebo	*P*	Mean difference (95% CI)	Odds ratio (95% CI)
Rohani et al. [34]	Iran	Outpatient	Hospitalization	Oral vitamin A, 25,000 IU/d and standard treatment for 10 d (89)	Glycerin tablet (91)	Proportion (*n*)	8 vs. 11	>0.05	0.72 (0.27, 1.88)	
			CRP (mg/dL)			Mean ± SD	3.4 ± 3.9 vs. 5.8 ± 9.7	0.039	2.40 (0.13, 4.67)	
Somi et al. [35]	Iran	Inpatient	Hospitalization duration (d)	Intramuscular vitamin A, 50,000 IU/d for a maximum for 2 wk (15)	Common treatment protocol (15)	Mean ± SD	7.33 ± 2.31 vs. 6.78 ± 1.84	0.49	−0.55 (−2.11, 1.01)	
			Mortality			Proportion (*n*)	3 vs. 2	0.68		1.63 (0.23, 11.46)
			Invasive mechanical ventilation			Proportion (*n*)	3 vs. 3			

Abbreviations: CRP, C-reactive protein.

^1^Hydroxychloroquine, antiviral regimen, corticosteroid, and antibiotics.

**TABLE 3:** Summary of studies—vitamin C interventions

Study ID	Country	Setting	Outcomes	Treatment arm (*n*)	Control arm (*n*)	Statistic	Treatment vs. placebo	*P*	Effect measure^1^
Coppock et al. [38]	United States	Inpatient	Clinical improvement within 72 h	IV vitamin C, 0.3 g/kg every 2 h, 0.6 g/kg on first day, 0.9 g/kg/d for 4 d, and standard of care defined as routine clinical care (44)	Standard treatment (22)	Proportion (*n*)	16 vs. 38	0.158	
						Odds ratio (90% CI)			2.36 (0.66, 8.07)
			Time to 50% reduction in supplemental oxygen (d)			Mean^1^	1.87 vs. 2.24	0.334	
						Hazard ratio (90% CI)			1.13 (0.65, 1.97)
			Time to discharge (d)			Mean	4.30 vs. 4.65	0.453	
						Hazard ratio (90% CI)			1.03 (0.61. 1.76)
Fogleman et al. [37]	United States	Outpatient	Symptom score (survey data)	Oral vitamin C, 1000 mg/d for 14 d (32)	Cornstarch capsule (34)	Mean ± SD	Day 14: 4.42 ± 4.48 vs. 3.38 ± 4.09 Day 30: 3.87 ± 9.72 vs. 2.21 ± 3.57	0.33 0.36	−1.04 (−3.15, 1.07) −1.66 (−5.22, 1.90)
JamaliMoghadamSiahkali et al. [22]	Iran	Inpatient	Hospitalization (d)	IV vitamin C, 1.5 g every 6 h and control arm for 5 d (30)	Lopinavir/ritonavir; hydroxychloroquine twice daily (30)	Median (IQR)	8.50 (7.0–12.0) vs. 6.50 (4.0–12.0)	0.028	1.75 (0.77–2.73)
			ICU (d)				5.50 (5.0 – 10.0) vs. 5.0 (5.0 – 7.0)	0.381	1.00 (0.43–1.57)
			Intubation			Proportion (*n*)	5 vs. 4	>0.9	1.30 (0.31–5.40)
			Mortality				3 vs. 3	>0.9	1.00 (0.22–4.56)
Kumari et al. [19]	Pakistan	Inpatient	Recovery (d)	IV vitamin C, 50 mg/kg/d and control arm (75)	Antipyretics, dexamethasone, prophylactic antibiotics (standard therapy) (75)	Mean ± SD	7.1 ± 1.8 vs. 9.6 ± 2.1	<0.0001	−2.50 (−3.13, −1.87)
			Hospitalization (d)				8.1 ± 1.8 vs. 10.7 ± 2.2	<0.0001	−2.60 (−3.24, −1.96)
			Mechanical ventilation			Proportion (*n*)	12 vs. 15	0.406	0.76 (0.33, 1.76)
			Mortality (overall)				7 vs. 11	0.31	0.60 (0.22, 1.64)
Labbani-Motlagh et al. [33]	Iran	Inpatient	Clinical improvement (NEWS score)	IV vitamin C, 12 g/d for 4 d (37)	Dextrose (37)	Mean ± SD	Day 3: 6.02 ± 0.43 vs. 5.20 ± 0.43 Day 5: 5.51 ± 0.46 vs. 5.04 ± 0.46	0.18 0.47	0.82 (−0.38, 1.75) 0.47 (−0.80, 1.75)
			CRP (mg/L)				Day 3: 45.78 ± 7.02 vs. 51.44 ± 7.28 Day 5: 35.29 ± 6.91 vs. 35.84 ± 7.42	0.58 0.98	−0.55 (−20.43, 19.33)
			Hospitalization (d)				9.24 ± 7.50 vs. 8.19 ± 5.34	0.52	−1.05 (4.92 – 2.18)
			ICU (d)				1.95 ± 5.89 vs. 1.51 ± 4.25	0.72	0.44 (−2.81, 1.95)
Majidi et al. [23]	Iran	Inpatient	Survival duration postintervention (d)	Oral vitamin C, 500 mg/d for 14 d (31)	Enteral nutrition (69)	Mean^3^	8 vs. 4	**<**0.01	
			Survival 2 wk postintervention (*n*)			Proportion (%)	16.1 vs. 2.9	0.028	6.44 (1.18, 35.31)^2^
Tehrani et al. [36]	Iran	Inpatient	CRP (mg/L)	IV vitamin C, 2 g every 6 h for 5 d and standard of care (18)	Standard treatment (26)	Mean ± SD	29 ± 15 vs. 29 ± 22	0.52	0.00 (−10.93, 10.93)
			Hospitalization (d)				14 ± 8 vs. 17 ± 8	0.23	−3.00 (−7.81, 1.81)
			Mortality			Proportion (*n*)	0 vs. 4	0.21	0.16 (0.01, 2.76)^2^
Zhang et al. [25]	China	Inpatient	IMV free days in 28 d	IV vitamin C, 24 g/d for 7 d (27)	Bacteriostatic water (29)	Median (IQR)	26.0 (9.0–28.0) vs. 22.0 (8.5–28.0)	0.57	1.3 (−4.7, 7.2)
			CRP (mg/L)				Day 3: 43.5(3.4–65.7) vs. 66.3 (29.8–107.4) Day 7: 29.5 (11.0–110.9) vs. 30.2 (2.3–131.7)	0.28 0.68	−4.8 (−68.1, 58.5) −12.6 (−75.3, 50.1)
			IL-6				Day 3: 113.1 (21.8–288.7) vs. 37.2 (5.6–85.3) Day 7: 19.4 (10.6–29.2) vs. 158.0 (15.3–259.6)	0.07 0.04	92.4 (−25.1, 210.0) −165.8 (−301.7, −29.8)
			Symptom improvement			Proportion (*n*)	5 vs. 6	0.84	0.90 (0.20, 3.3)^2^
			ICU (d)			Mean ± SD	22.9 ± 14.8 vs. 17.8 ± 13.3	0.20	5.10 (−2.58, 12.78)
			Hospitalization (d)				35.0 ± 17.0 vs. 32.8 ± 17.0	0.65	2.20 (−7.08, 11.48)
			Mortality (hospital)			Proportion (*n*)	6 vs. 11	0.20	0.47 (0.14, 1.52)
			28-d mortality				6 vs. 10	0.31	0.54 (0.17, 1.78)

Abbreviation: IMV, invasive mechanical ventilation.

1 Mean difference (95% CI).

2 Odds ratio (95% CI).

3 SD not reported.

**TABLE 4:** Summary of studies—combination treatments

Study ID	Country	Setting	Outcomes	Treatment arm (*n*)	Control arm (*n*)	Statistic	Treatment vs. placebo	*P*	Effect measure
Mahjoub et al. [42]	Tunisia	Outpatient	Symptom clearance	Multivitamin pill^1^, melatonin, and zinc (82)	Magnesium stearate, 3.5 mg Microcrystalline cellulose, 346.5 mg (82)	Day 10, Proportion (*n*)	66 vs. 55	0.038	2.03 (0.99, 4.14)^2^
			Symptom clearance			Day 30, Proportion (*n*)	82 vs. 81	0.316	3.04 (0.12, 75.6)^2^
Majeed et al. [7]	India	Inpatient	Hospitalization (d)	500 mg capsule containing curcuminoids100 mg, andrographolides 50 mg, resveratrol 50 mg, zinc 10 mg, selenium 40 μg, and piperine 3 mg (45)	Microcrystalline cellulose 500 mg (47)	Mean ± SD	7.41 ± 1.79 vs. 7.74 ± 2.35		
			Days to negative RT-PCR			Mean ± SD	7.43 ± 2.11 vs. 7.89 ± 3.68		
			Symptom severity			Mean ± SD	4.98 ± 2.18 to 1.36 ± 1.71 vs. 5.11 ± 2.29 to 1.70 ± 1.68	0.3413	
Thomas et al. [24]	United States	Outpatient	Time to 50% reduction in symptoms (d)	Zinc gluconate, 50 mg and vitamin C, 8000 mg for 10 d (58)	Standard of care (50)	Mean ± SD	5.5 ± 3.4 vs. 6.7 ± 4.4		−1.3 (−2.86, 0.32)
			Hospitalization			Proportion (*n*)	7 vs. 3		2.15 (0.53, 8.80)^2^
			Mortality			Proportion (*n*)	2 vs. 0	0.40	4.32 (0.20, 92.0)^2^
Ried et al. [31]	Turkey	Inpatient	Symptom free, hospital discharge	IV Vitamin C + HCQ + zinc + AZM + vitamin D3 (162)	HCQ + zinc + AZM + vitamin D3 (75)	Day 15, Proportion (*n*)	93 vs. 29	0.0069	2.14 (1.22, 3.74)^2^
			Symptom free, hospital discharge			Day 45, Proportion (*n*)	68 vs. 46		0.46 (0.26, 0.80)^2^
Yang et al. [39]	China	Inpatient	Disease recovery time (d)	Western medicine, traditional Chinese medicine, and high-dose vitamin C (10)	Western medicine and traditional Chinese medicine (10)	Mean ± SD: severely infected patients	13.45 ± 11 vs. 15.89 ± 4.1	<0.05	2.44 (−5.35, 10.23)
			Symptom clearance time (d)			Mean ± SD: severely infected patients	10.2 ± 1.75 vs. 13.4 ± 2.76	<0.05	3.20 (1.03, 5.37)
Kumar et al. [41]	India	Outpatient	CRP (mg/L)	APMV2020^3^ twice a day for 10 d (99)	Standard of care (93)	Mean difference ± SD	5.26 ± 13.6 vs. 4.52 ± 7.48	0.772	
Abulmeaty et al. [27]	Saudi Arabia	Inpatient	CRP (mg/L)	Nutritional supplement capsule^4^ for 10 d (24)	Cellulose capsule, 0.3 g (20)	Mean ± SD	12.8 ± 11.9 vs. 14.4 ± 10.5	0.603	1.6 (−5.29, 8.50)
			IL-6 (pg/mL)			Mean ± SD	8.91 ± 4.23 vs. 11.89 ± 1.62	0.035	2.98 (0.95, 5.00)
Hakamifard [32]	Iran	Inpatient	ICU admission	Oral vitamin C, 1000 mg; oral vitamin E, 400 IU; standard of care daily (38)	Standard of care (34)	Proportion (*n*)	3 vs. 5	0.380	0.49 (−0.11, 2.26)^2^
			Clinical improvement			Proportion (*n*)	35 vs. 29		2.01 (0.44, 9.14)^2^
			Hospitalization (d)			Mean ± SD	7.95 ± 3.18 vs. 8.03 ± 2.83	0.821	0.08 (−1.34, 1.5)
Darban et al. [21]	Iran	Inpatient	CRP (mg/L)	IV vitamin C 2 g; oral melatonin 6 mg; oral zinc sulfate 50 mg every 6 h for 10 d; standard of care (10)	Standard of care (10)	Mean ± SD	4.8 ± 3.6 vs. 4.4 ± 3.1	0.06	−0.40 (−3.56, 2.76)
			ICU (d)			Mean ± SD	14.1 ± 4.2 vs. 15 ± 3.3	0.30	0.90 (−2.65, 4.45)
Beigmohammadi et al. [20]	Iran	Inpatient	IL-6	Vitamins A, B, C, D, and E^5^ for 7 d (30)	No vitamins or placebo (30)	Mean ± SD	86.8 ± 102.5 vs. 130.2 ± 254.3	0.003	43.4 (−56.8, 143.6)
			CRP			Mean ± SD	34 ± 37.16 vs. 93.07 ± 56.08	0.001	59.1 (34.5, 83.7)
			Hospitalization for >7 d			Proportion (*n*)	4 vs. 16	0.002 0.247	Unadjusted: 0.135 (0.038, 0.481)^2^ Adjusted: 0.402 (0.086, 1.883)^2^
			Mortality			Proportion (*n*)	0 vs. 4	0.112	0.097 (0.005, 1.88)^2^
Hellou et al. [40]	Israel	Inpatient	Clinical improvement (NEWS2 score)	ArtemiC spray, 1 mL/10 puffs twice a day; and standard of care for 15 d or discharge (33)	Solvent spray and standard of care (17)	Mean ± SD	0.52 ± 0.67 vs. 2.23 ± 3.20	0.042	1.71 (0.55, 2.87)
			Supplemental oxygen use			Proportion (*n*)	7 vs. 5	0.728	0.65 (0.17, 2.46)^2^
Reino-Gelardo et al. [30]	Spain	Inpatient	Reduction in length of hospital stay	Food supplement (probiotics, prebiotics, vitamin D, zinc, and selenium) (78)	No food supplement (84)	Day 5, recovery (%)	92.6 vs. 41.2	0.006	
			Duration of gastrointestinal symptoms			Days	2.6 ± 1.3 vs. 4.3 ± 2.2	0.001	

Abbreviations: AZM, azithromycin; HCQ, hydroxychloroquine; IL, interleukin; ICU, intensive care unit.

ArtemiC 1 mL/10 puffs amounts to total daily dose of 12 mg artemisinin, 40 mg curcumin, 30 mg frankincense, and 120 mg vitamin C.

1 Multivitamin pill contained 10 μg provitamin A; vitamin B (0.65 mg vitamin B-1, 0.7 mg vitamin B-2, 8 mg vitamin B-3, 3 mg vitamin B-5, 0.7 mg vitamin B-6, 25 μg vitamin B-8, 100 μg vitamin B-9, and 1.25 μg vitamin B-12); 40 mg vitamin C; 2.5 μg vitamin D; 6 mg vitamin E; and minerals.

2 OR (95% CI).

3 Aspirin, 150 mg; promethazine hydrochloride, 5 mg; vitamin D3, 2000 IU; vitamin C, 750 mg; niacinamide, 80 mg; zinc, 15 mg; potassium iodide, 100 μg; and sodium selenate, 82.5 μg.

4 Vitamin A, 1500 μg; vitamin C, 250 mg; vitamin E, 90 mg; zinc, 7.5 mg; and selenium, 15 μg.

5 5000 IU daily of vitamin A, 600,000 IU once during the study of vitamin D, 300 IU twice daily of vitamin E, 500 mg 4 times daily of vitamin C, and 1 amp daily of vitamin B complex for 7 d.

### Risk of bias

**FIGURE 2:**
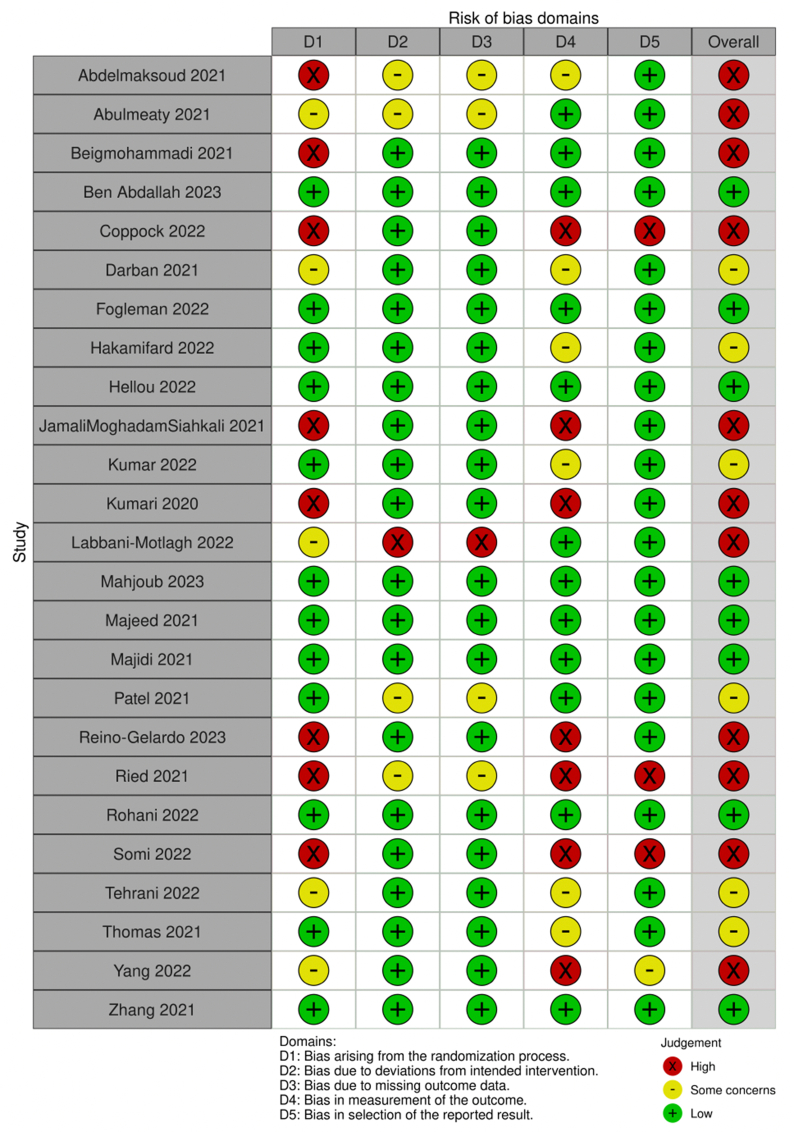
Risk of bias summary based on Cochrane Systematic Review Guidelines for included randomized controlled trials.

**FIGURE 3:**
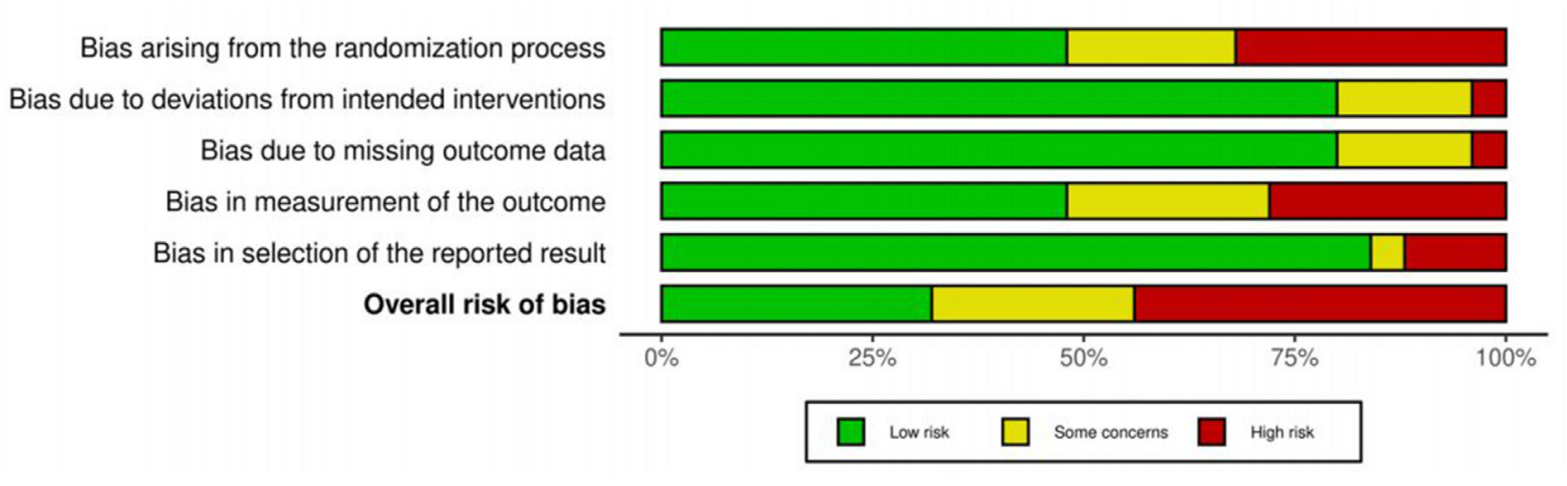
Summary of risk of bias assessment.

An overall risk of bias was determined from the assessments. Eight studies were identified as having low bias [[7], [23],^25^,^34^,^37^,^40^,^42^,^43^], whereas 6 studies were deemed to have some concerns [21,24,29,32,36,41]. The remaining 11 studies were classified as low-quality owing to high bias (Figure 2) [19,20,22,[26], [27],^30^,^31^,^33^,^35^,^38^,^39^]. A detailed summary that includes the assessment of individual domains and the overall risk of bias for each study was compiled and is presented in Figure 3 [17].

### Zinc interventions

The dosage of zinc provided in the trials ranged from 0.24 mg/d per kilogram of weight to 50 mg over 7–15 d or until clinical recovery. A comprehensive summary of these studies is presented in Table 1.

Abdelmaksoud et al. [26] evaluated the effect of 50-mg zinc twice daily, in comparison with standard treatment, in the treatment of inpatients with SARS-CoV-2 infection until their recovery. The study indicated that the mean recovery time for olfactory and/or gustatory dysfunction was significantly shorter in patients who received zinc therapy than that in those who did not (7 compared with 18 d; *P* < 0.001) [26]. No impact was observed on the overall duration of recovery from COVID (*P* > 0.05) [26]. Another study explored the effect of 25-mg zinc twice daily compared with a placebo over 15 d in both inpatients and outpatients [43]. Among inpatients, significant differences were observed in ICU admission rates and the length of hospital stay [43]. Specifically, fewer patients in the zinc intervention group required ICU admission than those in the control group (OR: 0.42; 95% CI: 0.21, 0.87), and their mean hospital stay was 3.5 d shorter (*P* < 0.001) [43]. The study also reported mortality rates, although this difference was not statistically significant (OR: 0.68; 95% CI: 0.34, 1.35) [43]. For outpatients, the zinc group experienced shorter symptom duration by an mean of 1.9 d (0.62–2.6) and a lower hospital admission rate (1.20% compared with 3.80%; OR: 0.30; 95% CI: 0.03, 2.8) [43]. A study by Patel et al. [29] investigated the administration of a high dose of intravenous (IV) zinc at 0.24 mg/kg/d for 1 wk or until hospital discharge or death. All participants were found to be zinc deficient at the baseline, with mean serum zinc concentrations indicating deficiency in both the zinc (7 ± 1.6 μmol/L) and placebo (6.9 ± 1.1 μmol/L) groups [29]. The zinc therapy group showed an increase in serum zinc concentrations to 10.77 μmol/L, above the zinc deficiency cutoff [29]. On the seventh day, there were fewer nonventilated patients requiring oxygen in the zinc group compared with those in the placebo group, although the difference was not statistically significant (*n* = 6 compared with *n* = 2; *P* = 0.19) [29].

### Vitamin A interventions

Oral vitamin A dosage ranged from 20,000 over 10 d to 200,000 IU over 2 d. Vitamin A was also delivered intramuscularly (Table 2).

An outpatient study by Rohani et al. [34] administered 25,000 IU/d of oral vitamin A compared with placebo to patients over a 10-d period. Both groups also received standard SARS-CoV-2 treatment. The study reported slightly lower hospitalization rates in the vitamin A group than those in the placebo group (9% compared with 12%), but this difference was not statistically significant [34]. However, the vitamin A group had significantly lower concentrations of CRP, a marker of inflammation (3.4 ± 3.9 compared with 5.8 ± 9.7 mg/dL; *P* = 0.039) [34]. In contrast, a study by Somi et al. [35], involving inpatients, receiving the intramuscular vitamin A (50,000 IU/d) for ≤2 wk, found a slightly longer hospital stay in the vitamin A group than that in the placebo group (7.33 ± 2.31 compared with 6.78 ± 1.84 d), but this difference was not statistically significant (*P* = 0.49) [35]. Both groups had similar rates of invasive mechanical ventilation [35]. The study also found that the odds of mortality were 1.63 times higher in the placebo group, although this was not statistically significant (95% CI: 0.23, 11.46; *P* = 0.68) [35].

### Vitamin C interventions

The studies examining vitamin C interventions used various methods of delivery, including both oral [23,37] and IV [19,22,25,33,36,38] routes, as detailed in Table 3. For oral administration, the dosage of vitamin C varied from 500 to 1000 mg/d over a period of 2 wk. Regarding IV delivery, the dosage ranged from 0.3 g administered every 2 h, per kilogram of weight, to a high of 24 g/d, administered for 4–7 d.

Studies on oral vitamin C supplementation by Fogleman et al. [37] and Majidi et al. [23] administered 1000 and 500 mg/d, respectively. Fogleman et al. [37] found no significant difference in symptom severity between the vitamin C and placebo groups, whereas Majidi et al. [23] observed a significantly longer survival duration postintervention (8 compared with 4 d; *P* < 0.001) and higher survival rate (16.1% compared with 2.9%; *P* = 0.028) in the vitamin C group than those in the placebo group [23].

IV vitamin C studies also showed mixed results [22,25,38]. Coppock et al. [38] investigated the effects of a dose of 0.3 g/kg every 2 h, increasing to 0.9 g/kg/d for 4 d in 44 hospitalized patients with SARS-CoV-2 infection, compared with 22 receiving standard treatment [38]. The study found no significant improvement in clinical symptoms (odds of improvement 90% CI: 0.66, 8.07; *P* = 0.158) and time to discharge (4.30 compared with 4.65 d; *P* = 0.453) [38]. However, another study [22] noted a shorter median hospital stay (8.50 compared with 6.50 d; *P* = 0.028) following 1.5 g of IV vitamin C every 6 h for 5 d in inpatients, than that after placebo [22]. Similarly, another trial assessed the impact of IV vitamin C in 75 patients receiving 50 mg/kg/d IV vitamin C compared with 75 patients on standard treatment and showed a significantly shorter recovery time (7.1 compared with 9.6 d; *P* < 0.0001) and hospitalization duration (8.1 compared with 10.7 d; *P* < 0.0001) following vitamin C intervention [19]. In contrast, Labbani-Motlagh et al. [33] assessed the effect of 12 g/d IV vitamin C for 4 d compared with a dextrose placebo and reported no significant differences in clinical improvement, hospitalization and ICU stay durations, or CRP concentrations [33]. Similarly, Tehrani et al. [36] administered 2 g of IV vitamin C every 6 h for 5 d hospitalized patients with SARS-CoV-2 infection compared with standard treatment as a control and found no significant differences in CRP concentrations, hospitalization duration, or mortality rates [36]. Finally, Zhang et al. [25] administered 24 g/d IV vitamin C for 7 d to 27 patients, comparing with 29 patients receiving bacteriostatic water, and observed no significant differences in most outcomes, including IL-6 concentrations and symptom improvement, except for IL-6 concentrations on day 7, which showed a significant decrease in the treatment group (*P* = 0.04) [25].

### Combined treatments

The studies on combined treatments investigated a variety of multivitamins, antioxidants, and minerals, with various comparator groups, as detailed in Table 4. Abulmeaty et al. [27] administered nutritional supplement capsules (contained 1500-μg vitamin A, 250-mg vitamin C, 90-mg vitamin E, 7.5-mg zinc, and 15-μg selenium) or a placebo to inpatients with SARS-CoV-2 infection for 10 d. The results suggest a significant dampening of the cytokine storm (*P* = 0.035) with improvements in some clinical parameters, such as lower body temperatures (*P* = 0.004), along with respiratory rate (*P* = 0.020) after 10 d [27]. Likewise, Majeed et al. [7] explored the use of a capsule, ImmuActive (contained 10-mg zinc and 40-μg selenium), or a placebo for inpatients with SARS-CoV-2 infection for 28 d and found no significant in hospitalization duration, symptom severity, and viral clearance. Mahjoub et al. [42] administered a daily combination of a multivitamin pill (contained provitamin A, vitamin B complex, along with vitamins A, C, D, and E), 2-mg melatonin, and 25-mg zinc, compared with placebo in mild-to-moderate SARS-CoV-2 cases. The study showed a significantly higher symptom clearance in the intervention group (80.5% compared with 67.1%; *P* = 0.038) by day 10, although this difference was not maintained by day 30 (*P* = 0.316) [42]. Ried et al. [31] investigated the effectiveness of the Zelenko protocol (which includes hydroxychloroquine, zinc, and azithromycin), combined with intravenous vitamin C (50 mg/kg every 6 h on day 1 and 100 mg/kg every 6 h for 7 d) and vitamin D3 (5000 IU/d), as opposed to the Zelenko protocol with only vitamin D3 (5000 IU/d) for SARS-CoV-2–positive inpatients [31]. This study highlighted that the addition of IV vitamin C contributed to quicker patient recovery (15 compared with 45 d; *P* = 0.006) [31]. Another study [30] explored the use of a food supplement containing probiotics, prebiotics, vitamin D (0.75 μg), zinc (1.5 mg), and selenium (8.25 μg) to reduce gastrointestinal symptoms and hospital stay duration in patients with SARS-CoV-2 infection [30]. The study demonstrated a reduction in the duration of gastrointestinal symptoms from 4.3 ± 2.2 d to 2.6 ± 1.3 d (*P* = 0.001) and faster recovery after 5 d of hospitalization (93.6% compared with 41.2%; *P* = 0.006) following the intervention [30].

No studies reported any serious adverse events. Only 1 study reported on the occurrence of hypersensitivity to vitamin C [33]. A few studies that explored combined treatments presented adverse events including diarrhea, nausea, and vomiting or stomach cramps [24,31].

### Meta-analysis

**FIGURE 4:**
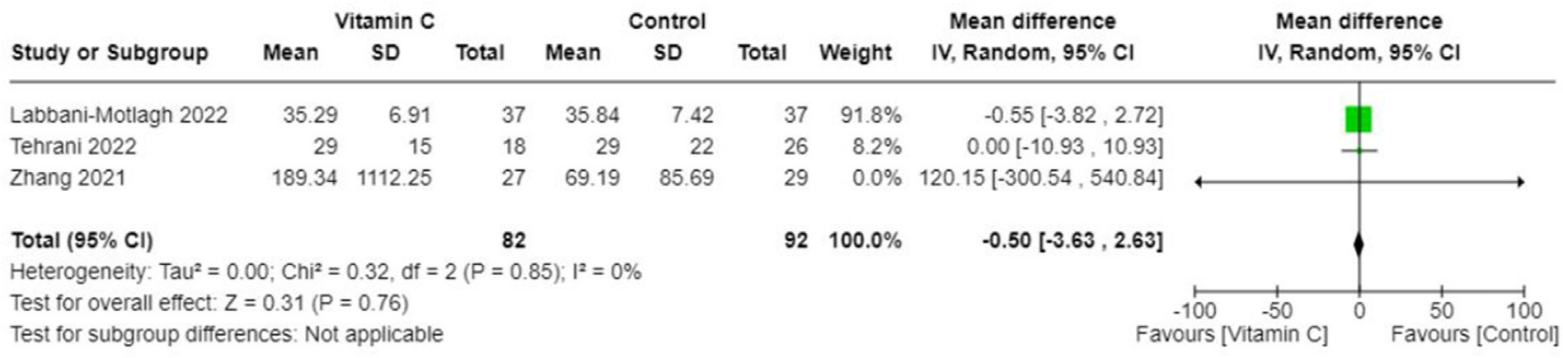
Change in CRP (mg/L) with vitamin c vs. placebo. CRP, C-reactive protein.

**FIGURE 5:**
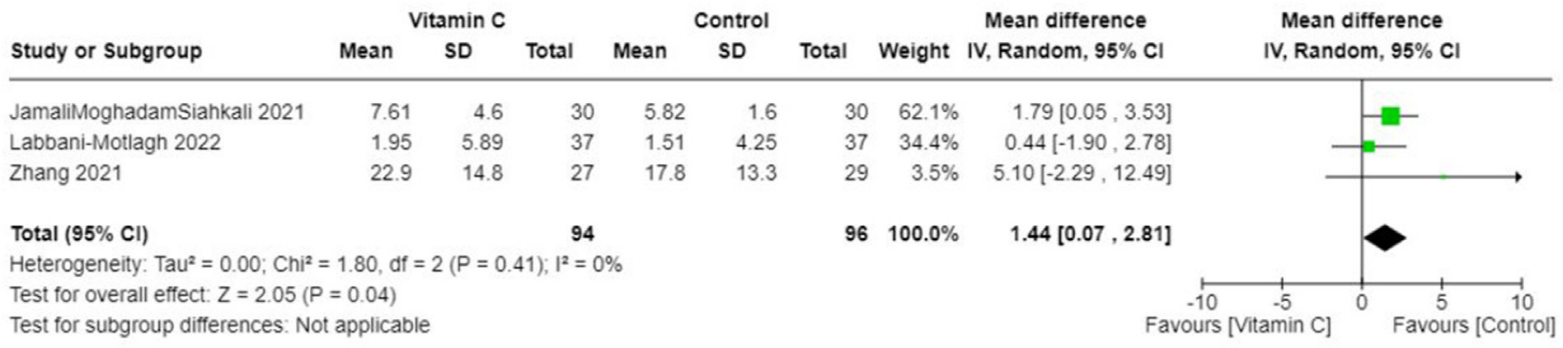
Duration in ICU (days) with vitamin C vs. placebo. ICU, intensive care unit.

**FIGURE 6:**
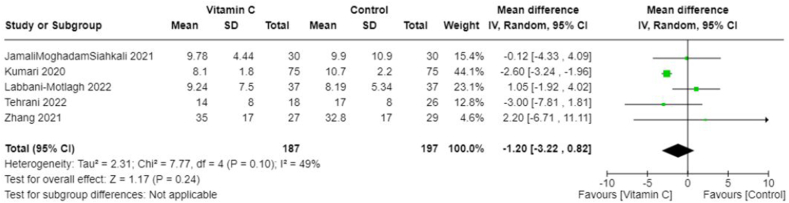
Duration in hospital (days) with vitamin C vs. placebo.

**FIGURE 7:**
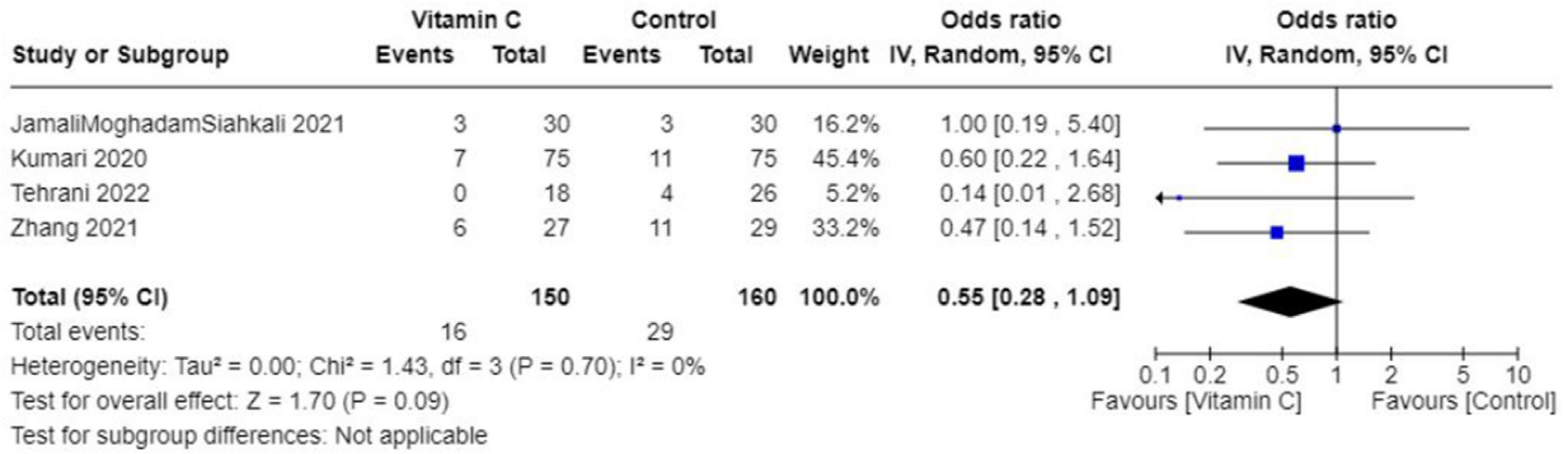
Mortality (proportion of patients) with vitamin C vs. placebo.

Owing to the heterogeneity outcomes of interest as well as in the types and combinations of treatments used in different studies, we were limited in our ability to perform a comprehensive meta-analysis across all antioxidant types. This was particularly true for interventions other than vitamin C. In the case of vitamin C studies, we found sufficient homogeneity in both the intervention type and the outcome measurements, which made these studies suitable for meta-analysis. All studies included in the meta-analyses involved IV administration of vitamin C. The pooled MD suggests no significant difference in CRP concentrations between those treated with vitamin C and those in the control group (−0.50; 95% CI: −3.63, 2.63; *I*^2^ = 0%; 3 studies [25,33,36], *n* = 174) (Figure 4), and there was no heterogeneity across studies. However, the overall effect favored vitamin C slightly, which meant that vitamin C seemed to reduce CRP concentrations compared with control. The pooled MD in the duration of days spent in the ICU suggests that the control group spent less time in the ICU compared with those treated with vitamin C (pooled MD: 1.44; 95% CI: 0.07, 2.81; *I*^2^ = 0%; 3 studies [25,33,36], *n* = 190) (Figure 5). Alternatively, patients treated with vitamin C spent fewer days in the hospital compared with those in the control group; however, this was not statistically significant (pooled MD: −1.20; 95% CI: −3.22, 0.82; *I*^2^ = 49%; 5 studies [19,22,25,33,36], *n* = 384) (Figure 6). Heterogeneity was minimal across studies in the analysis for duration in the ICU, whereas there was moderate heterogeneity in the studies that examined duration in the hospital. As for mortality, the pooled OR suggests that patients treated with vitamin C had lower odds of mortality than those in the control group (pooled OR: 0.55; 95% CI: 0.28, 1.09; *I*^2^ = 0%, 4 studies [19,22,25,36], *n* = 310) (Figure 7) with no heterogeneity across studies. However, this finding was not statistically significant.

**FIGURE 8:**
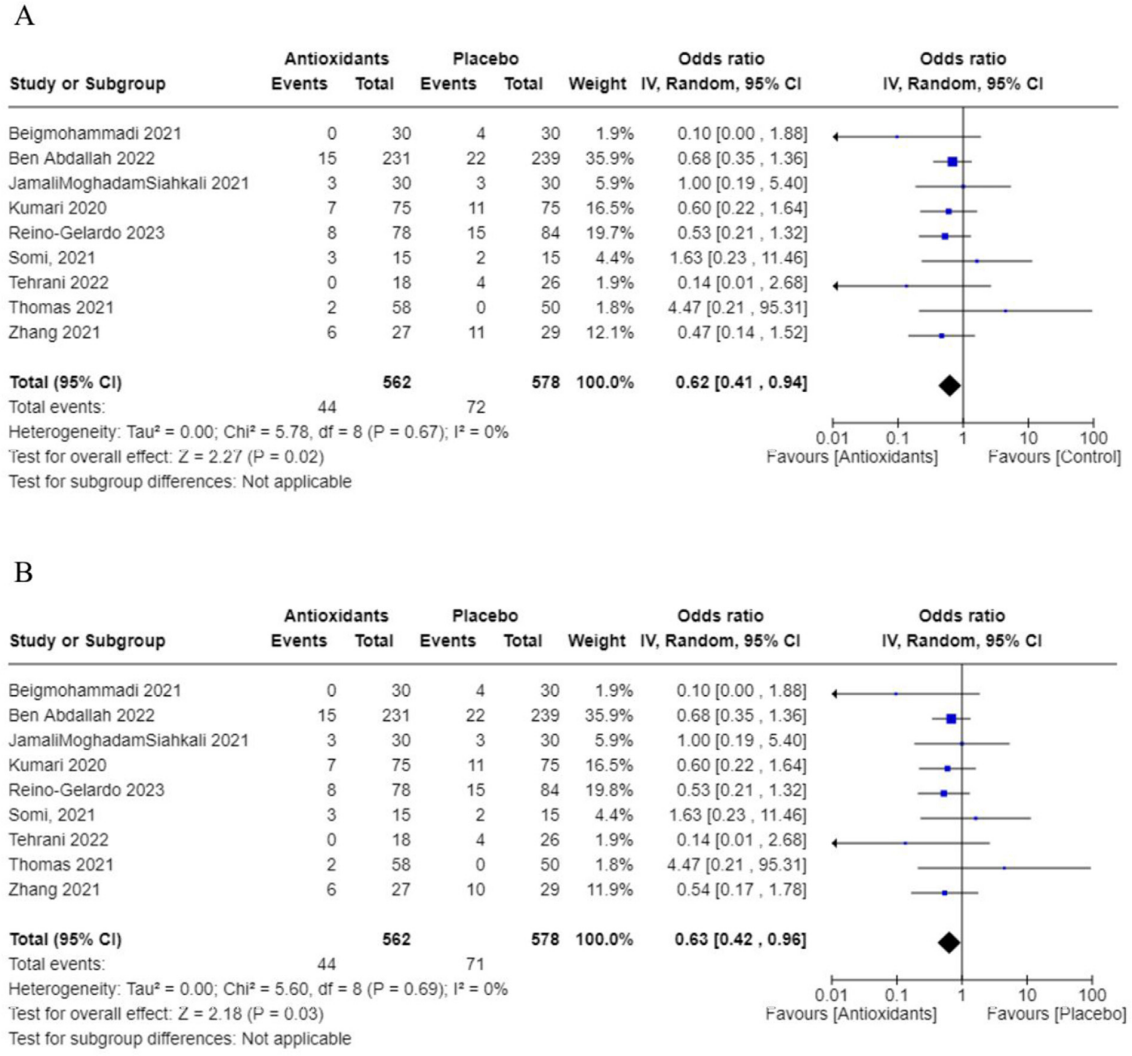
(A) Hospital mortality with antioxidants vs. placebo. (B) 28-d mortality with antioxidant vs. placebo.

**FIGURE 9:**
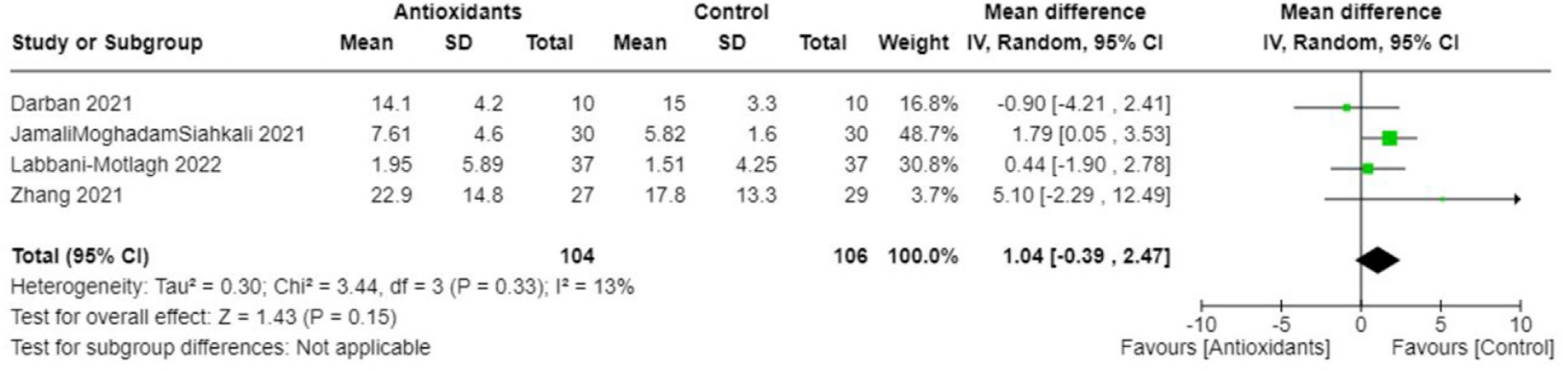
Duration of ICU stay (days) with antioxidant vs. placebo. ICU, intensive care unit.

**FIGURE 10:**
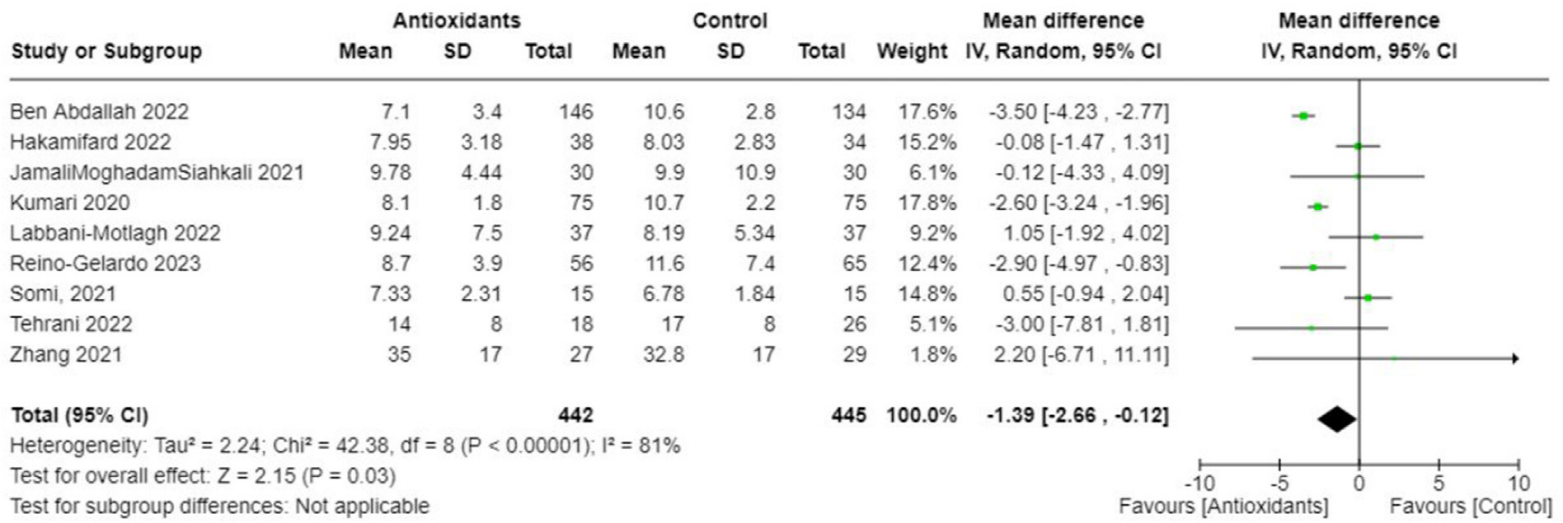
Duration of hospital stay (days) with antioxidant vs. placebo.

**FIGURE 11:**
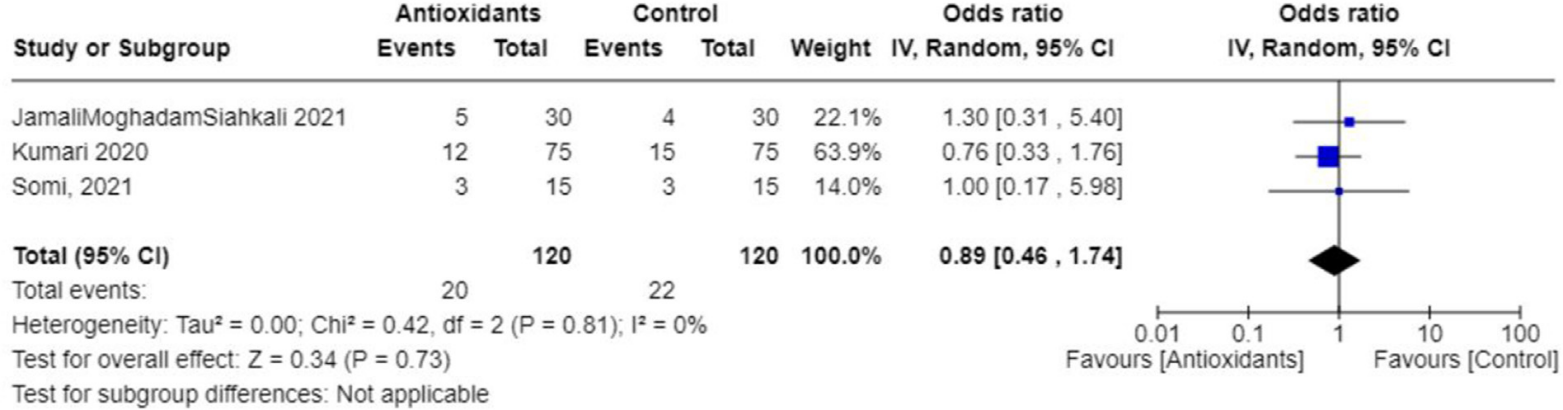
Need for artificial ventilation with antioxidant vs. placebo.

**FIGURE 12:**
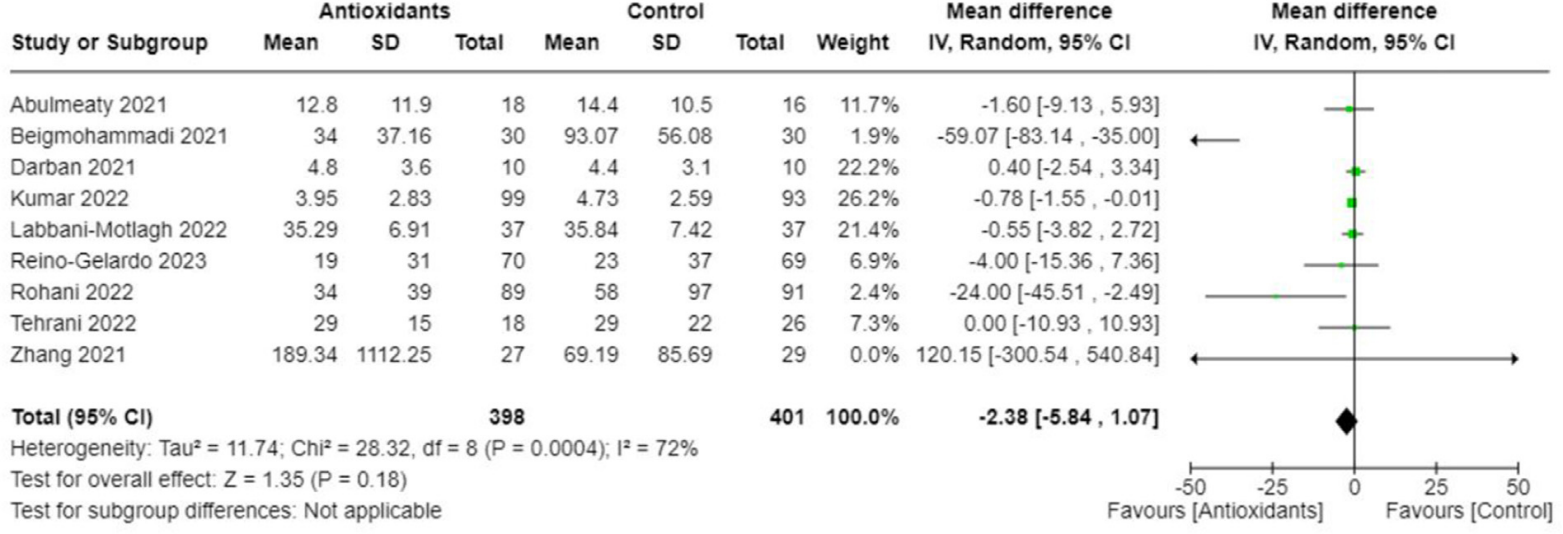
Changes in CRP (mg/L) with antioxidants vs. placebo. CRP, C-reactive protein.

**FIGURE 13:**
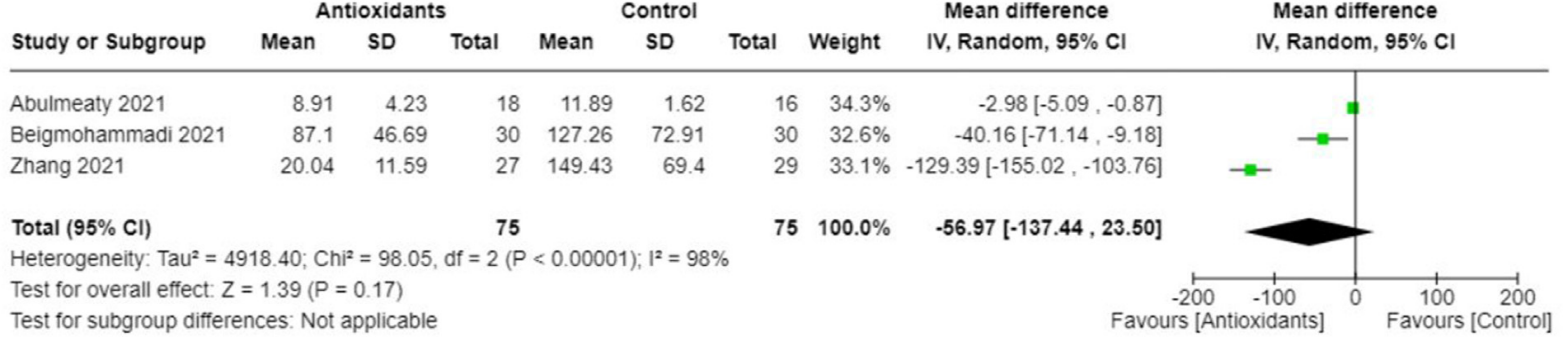
Changes in IL-6 (pg/mL) with antioxidants vs. placebo. IL, interleukin.

**FIGURE 14:**
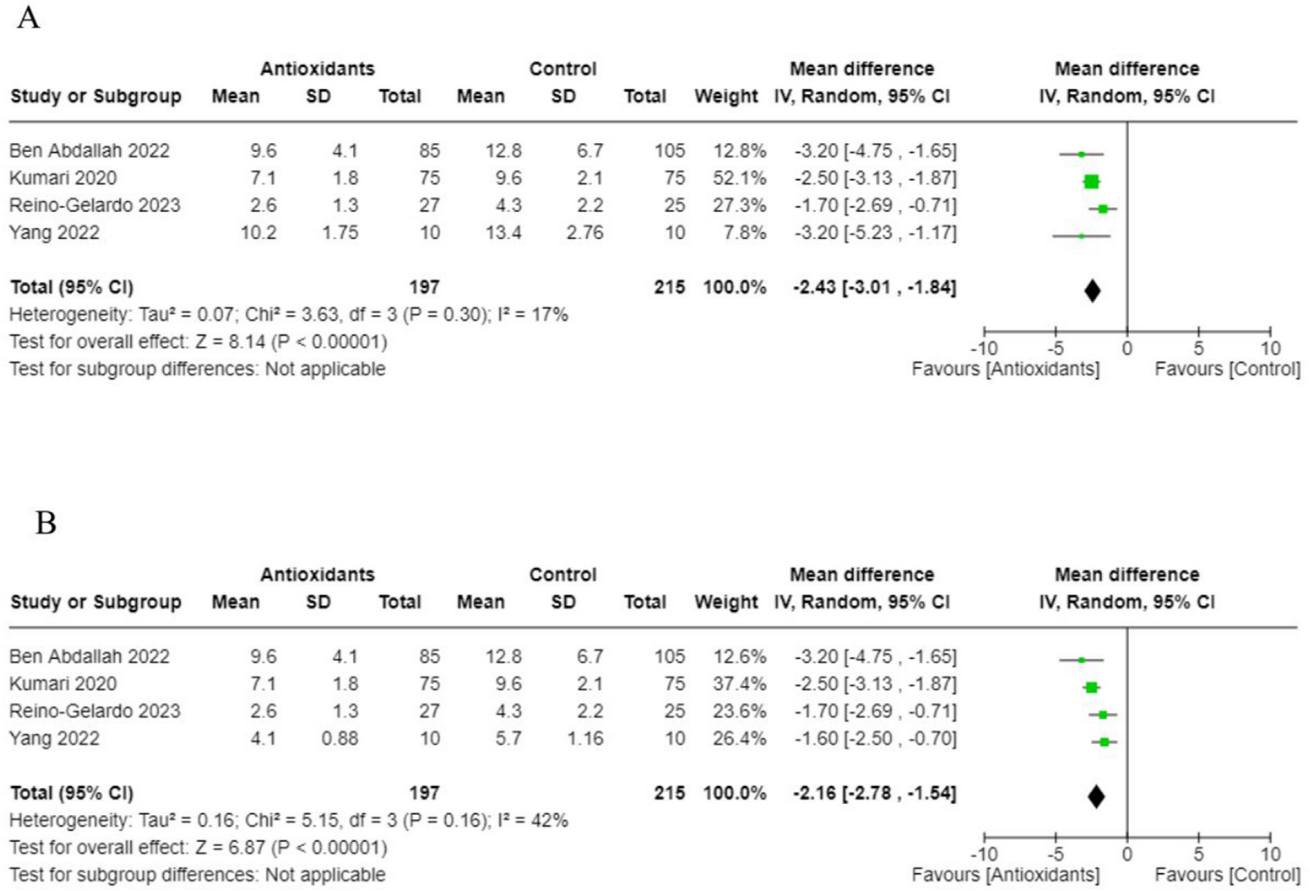
(A) Duration of symptoms (days) in severely infected patients with antioxidants vs. placebo. (B) Duration of symptoms (days) in mildly infected patients with antioxidants vs. placebo.

**FIGURE 15:**
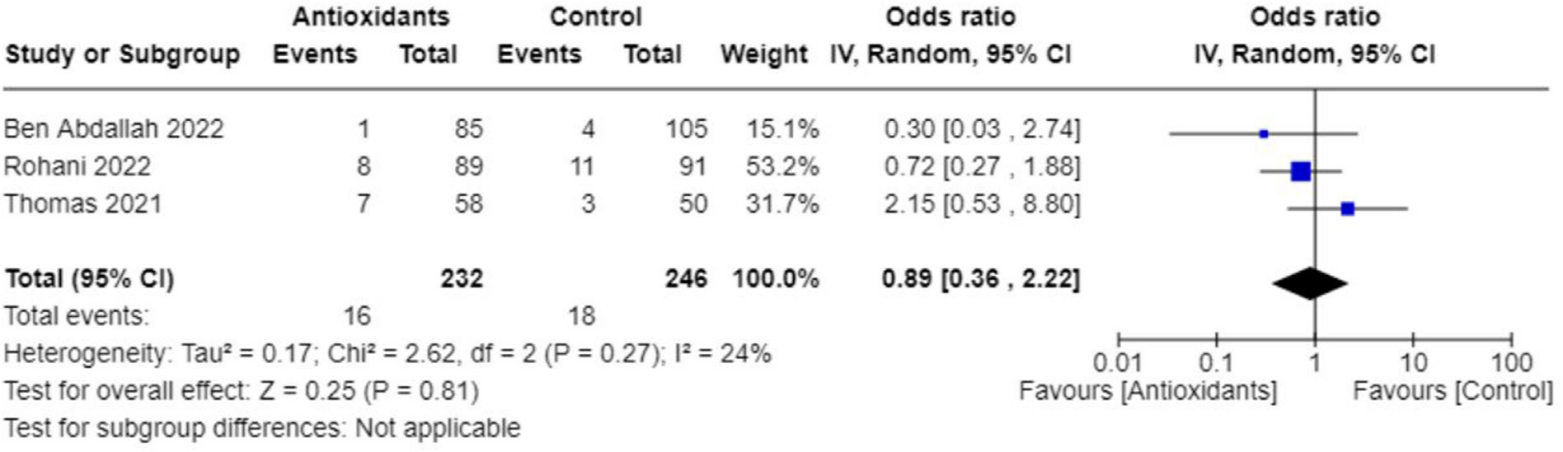
Hospital admission with antioxidants vs. placebo.

In addition, we performed meta-analyses combining interventions based on similar outcomes. Considering hospital mortality, pooled OR suggested that those treated with antioxidants had lower odds than those in the control group (0.62; 95% CI: 0.41, 0.94; *I*^2^ = 0%; 9 studies, *n* = 1140) (Figure 8). Days spent in the ICU did not differ between groups (pooled MD: 1.04; 95% CI: −0.39, 2.47; *I*^2^ = 13%; 4 studies, *n* = 210) (Figure 9); however, those in the antioxidant group had a shorter hospital duration (pooled MD: −1.39; 95% CI: −2.66, −0.12; *I*^2^ = 81%; 9 studies, *n* = 887) (Figure 10). The analyses on hospital duration had considerable heterogeneity. Additional outcomes such as need for artificial ventilation (Figure 11), changes in CRP concentrations (Figure 12), and changes in IL-6 concentrations (Figure 13) were comparable between the antioxidant and control groups, with heterogeneity varying from minimal to considerable heterogeneity. As for duration of symptoms, those in the antioxidant group experienced shorter duration than those in the control group (pooled MD: −2.43; 95% CI: −3.01, −1.84; *I*^2^ = 17%; 4 studies, *n* = 412) (Figure 14). Hospital admission was also similar between groups (pooled OR: 0.89; 95% CI: 0.36, 2.22; *I*^2^ = 24%; 3 studies, *n* = 478) (Figure 15).

A sensitivity analysis was initially planned to exclude low-quality studies, thereby focusing on only high-quality studies. However, owing to high risk of bias in 3 studies [19,22,33] and unclear risk of bias in 1 study [36], this analysis was not conducted in this review.

## Discussion

This systematic review and meta-analysis analyzed 25 peer-reviewed RCTs investigating the role of antioxidant therapies in the treatment and prognosis of SARS-CoV-2 infection. Interventions included zinc, vitamin A, vitamin C, and combined treatments comprising antioxidants, minerals, and/or multivitamins. Around 44% of the studies exhibited high risk of bias, and 24% presented some concerns regarding bias. These biases could be attributed to the challenges faced during the COVID-19 pandemic, such as modifications in research methodologies and difficulties in participant retention, potentially leading to attrition bias and impacting outcome measurement. Of the 25 studies included, only 5 met meta-analysis criteria owing to diverse methodologies and outcomes measurements. The meta-analysis revealed that IV vitamin C treatment did not significantly impact CRP concentrations or mortality rates among patients. Interestingly, the control group had a shorter ICU stay than those treated with IV vitamin C, but the inverse was true for the duration of hospitalization. The evidence indicates varied efficacy in antioxidant therapy for reducing SARS-CoV-2 severity. Despite all studies focusing on key outcomes such as recovery time, mortality, ICU and hospital stay durations, ventilation needs, changes in inflammatory biomarkers, symptom severity and duration, and the presence of long COVID, the variability in their findings prevents clear recommendations from being made at this stage.

In the context of existing literature, a previous systematic review of observational and intervention studies (*n* = 36) investigated the impact of antioxidants, such as vitamins C and D, selenium, and zinc, on key clinical outcomes in patients with SARS-CoV-2 infection [44]. The study noted improvements in inflammation, the Horowitz index, and mortality rates following vitamin C supplementation, with selenium and zinc influencing cure rates, ventilation requirements, ICU admissions, and disease complications. However, our meta-analysis, which focused exclusively on RCTs, did not observe these impacts with vitamin C. This discrepancy could be partly due to methodologic differences, as the previous review included a combination of 27 observational and 9 interventional studies. Yet, both studies underscore the necessity for well-designed large-scale trials to establish clear guidelines for antioxidants in SARS-CoV-2 treatment.

Focusing on individual antioxidants, zinc is known for its role in immune system development and function, which led to assumptions about its effectiveness in SARS-CoV-2 progression [45]. However, the studies reviewed present a mixed picture. Although some suggest benefits like shortened symptom duration and decreased ICU admissions [43], others indicate no significant change in overall recovery time [26]. This inconsistency warrants further validation before drawing firm conclusions about zinc’s benefits in COVID-19 treatment.

Similarly, vitamin A showed varying effects in the studies reviewed. Although no significant difference was observed in hospitalization rates [34,35], some studies did report lower CRP concentrations in vitamin A–treated patients [34]. CRP, an acute-phase protein, serves as an early marker of infection or inflammation [46]. Studies suggest that CRP concentrations are an early indicator of severity of SARS-CoV-2 infection [46]. Similar to zinc, more comprehensive studies are necessary to conclusively determine vitamin A’s efficacy in treating SARS-CoV-2 infection.

As for the studies that target vitamin C intervention, our findings revealed that the efficacy of vitamin C varied across different doses and methods of administration. The oral supplementation studies, such as those by Fogleman et al. [37] and Majidi et al. [23], which administered 1000 and 500 mg/d, respectively, presented mixed outcomes. Fogleman et al. [37] found no significant difference in symptom severity between the vitamin C and placebo groups, whereas Majidi et al. [23] reported a significant improvement in survival duration with vitamin C treatment compared with enteral nutrition. IV vitamin C administration studies demonstrated a similar variability. The study by Coppock et al. [38], who used a dosing regimen of ≤0.9 g/kg/d, did not show significant improvements in clinical symptoms or oxygen supplementation compared with standard treatment [38]. However, other studies that used higher doses of IV vitamin C observed notable benefits such as shorter hospital stays and faster recovery times [19,22]. Yet, these benefits did not extend to reductions in mechanical ventilation or mortality rates [19,22]. Further complicating the interpretation, studies by Labbani-Motlagh et al. [33] and Tehrani et al. [36], despite using high-dose IV vitamin C, found no significant differences in clinical improvement, CRP concentrations, hospitalization duration, or mortality rates when compared with control groups [33,36]. A distinct finding in the study by Zhang et al. [25] was the significant decrease in IL-6 concentrations with high-dose IV vitamin C, suggesting an anti-inflammatory effect of vitamin C [25]. Our meta-analysis integrated these varied findings and indicated no significant difference in CRP concentrations or mortality rates between those treated with vitamin C and control groups. However, a slight overall trend favoring vitamin C in CRP reduction was noted. Interestingly, the meta-analysis showed that patients in the control group had shorter ICU stays, whereas it was suggested that those treated with vitamin C experienced reduced hospitalization durations. Although the length of ICU stay may be impacted by the need for additional physiologic support, these results collectively suggest that although vitamin C, particularly in higher doses, may confer certain benefits in recovery time and hospitalization duration, its impact on mortality and ICU stay is less clear. The diversity in dosages and patient populations highlights the challenges in drawing definitive conclusions about vitamin C’s effectiveness in treating SARS-CoV-2 infection, highlighting the need for further research to better understand the potential therapeutic value.

Combined treatments involving antioxidants, multivitamins, and minerals showed varied results, largely owing to different combinations and dosages. Certain combinations, such as vitamins C and/or D with zinc, indicated potential benefits in early symptom clearance [30,31,42]. In contrast, studies exploring other combinations showed mixed results, suggesting that the specific components of the combination treatments play a critical role [20,27]. For example, Beigmohammadi et al. [20] included zinc and selenium but not vitamin B and D, whereas Abulmeaty et al. [27] who did include vitamins B and D reported differences in CRP concentrations, suggesting that these vitamins might be key contributors to reducing inflammation markers like CRP [20,27]. Despite differences, both studies consistently showed significant changes in IL-6 concentrations, another important inflammatory marker [20,27], highlighting complexity of determining the efficacy of combination treatments and the need for further research to explore the effects of various combinations and dosages of antioxidants in SARS-CoV-2 treatment.

In terms of antioxidant deficiencies, numerous studies have highlighted their potential impact on the disease state, referring to the severity and progression of COVID [[20], [21], [22], [23], [24], [25], [26],^29^,[31], [32], [33], [34], [35], [36], [37], [38], [39], [40], [41], [42], [43]]. However, only 1 study reported on antioxidant status at baseline and examined the changes following intervention [29]. Future studies should investigate the role of antioxidant supplementation in addressing deficiencies that may exist before illness or arise during the prognosis of COVID.

### Strengths and limitations

The strengths of this review include the broad systematic literature search, clearly defined inclusion and exclusion criteria, independent study selection, data extraction, methodologic quality assessment, and rigorous meta-analyses.

To our knowledge, this study provides the most comprehensive pooled data on the effects of antioxidant therapy on COVID-related outcomes. We acknowledge several limitations. Studies varied in intervention specifics such as dose, regimen, and duration. To mitigate this, we grouped studies with similar interventions and outcomes in the systemic literature review and meta-analysis, allowing for a more structured and coherent presentation of the findings. The findings of the review are limited to participants older than 18 y because no studies were found that were conducted on individuals younger than 18 y. Although this prevents the generalizability of the results to a younger demographic, it highlights an area for future research to address this gap in evidence. Given the rapidly evolving nature of COVID research, there is a possibility of publication bias toward studies with positive results. We attempted to mitigate this by assessing the methodologic quality of each study. Finally, the exclusion of studies based on language might introduce some bias; however, it is worth noting that there were only 2 non-English studies that were potentially eligible for inclusion. This suggests that the impact of language bias may be minimal in this particular review.

In the context of clinical application, this systematic review highlights the complex landscape of antioxidant therapies, such as zinc and vitamin C, for treating SARS-CoV-2 infection. Although some studies suggest potential benefits, there is no consensus or definitive evidence supporting the efficacy of these antioxidants. Our study is limited by the heterogeneity of populations, interventions, and outcome measures, along with inadequate sample size may contribute to nonsignificant trends and a tendency toward null findings. This inconsistency underscores the challenges in determining the role of antioxidant therapy in the clinical management of patients with SARS-CoV-2 infection. Despite these limitations, our findings suggest that zinc and vitamin C supplementation may shorten symptom duration and reduce hospital stay in patients with SARS-CoV-2 infection. These results are noteworthy given the need for effective interventions to manage health service capacity during the pandemic. Further well-designed large-scale clinical trials are warranted to confirm these findings and establish more definitive conclusions. Given the conflicting nature of previous findings, any interpretation of the benefits of antioxidant regimens must be approached with caution. The ambiguity in the current evidence highlights an urgent need for further research. As pharmaceutical companies and government institutions continue to explore various treatments, including antivirals, to combat SARS-CoV-2, it is imperative that they also acknowledge and invest in research exploring the potential, yet unclear, role of antioxidant therapy. This is especially pertinent as new variants emerge, which may present unique challenges to existing therapeutic approaches, including antivirals. In-depth, well-designed studies are required to ascertain the effectiveness of antioxidants in treating SARS-CoV-2, including their potential role in addressing variants and long-term symptoms.

## Conclusions

This systematic review and meta-analysis evaluated the effectiveness and safety of antioxidant therapy in combating COVID-19, synthesizing findings from various RCTs. Our findings revealed that some antioxidants, including vitamin A, vitamin C, and zinc, may offer some benefit in reducing SARS-CoV-2 complications, particularly when used in combination. These preliminary findings provide a basis for considering antioxidants in the management of the disease. However, it is crucial to note that many of the studies included in our review were characterized by high risk of bias, which raises concerns about the reliability of the reported benefits. This highlights the pressing need for more rigorously designed RCTs to evaluate the efficacy of antioxidant therapy in a more conclusive manner. Future studies should aim to minimize bias and improve the quality of evidence, which will be vital in updating clinical guidelines and informing therapeutic strategies. Given the ongoing challenges of long COVID and its impact on quality of life, understanding the potential role of antioxidants could be particularly valuable. By providing more definitive evidence on the role of antioxidants, future research could contribute to reducing the disease burden associated with long-term complications of SARS-CoV-2. Therefore, although our review suggests potential benefits of antioxidant therapy, the path to integrating these treatments into standard care remains contingent on further high-quality research.

## Author contributions

The author’s responsibilities were as follows – BH, ADP, DJ: conceived the work; CZ: conducted the literature search; RS, TO, AP, TV, LAP, KS: conducted the systematic review and drafted the manuscript; AP, LAP: edited and modified the manuscript; BH: supervised the conduct of this review from inception to analysis and modified the manuscript; and all authors: contributed to revising the manuscript for important intellectual content, provided final approval of the version to be published, and agreed to be accountable for all aspects of the work.

## Funding

This project was funded by research grants OTT-183092 and PPE-190332 funded by the Canadian Institutes of Health Research. The sponsors had no role in the study design, conduct, or interpretation of results.

## Data availability

Data described in the manuscript, code book, and analytic code will be made available on reasonable request.

## Conflict of interest

The authors report no conflicts of interest.
