# Supplementary material for: Role of Antioxidant Therapy in the Treatment and Prognosis of COVID-19: A Systematic Review and Meta-analysis of Randomized Controlled Trials
Source: Curr Dev Nutr. 2024 Mar 24;8(5):102145. doi: 10.1016/j.cdnut.2024.102145 (PMC11061685; doi:10.1016/j.cdnut.2024.102145)
Supplement: Multimedia component 1 [file mmc1.docx]

**Supplementary Table 1:** Study details

| Study ID | Location | Population | Sample size | Demographics | Intervention | Objective | Results |
| --- | --- | --- | --- | --- | --- | --- | --- |
| Abdelmaksoud 2021[26] | Egypt | Positive COVID-19 inpatients | 134 | Mean age 31.8 +/- 13.1 (mild), 47.8 +/- 15.8 (common), 59.1 +/- 9.5 (severe), 69.5 +/- 6.5 (extremely severe) | Zinc | To shed light on the possible role of zinc therapy regarding the improvement of impaired olfaction among patients with COVID-19 | Zinc therapy lowered duration of smell recovery without affecting total recovery duration. |
| Abulmeaty 2021[27] | Saudi Arabia | Positive COVID-19 inpatients | 52 | Mean age 45.08 +/- 9.19, 63.6% male | Vitamin A; Vitamin C; Vitamin E; Zinc; Selenium | Investigate the effect of antioxidant supplements on inflammatory cytokines and disease progression in non-critically ill COVID-19 patients | Intervention group significantly dampened inflammation and lead to partial improvements in clinical parameters. |
| Beigmohammadi 2021[20] | Iran | Positive COVID-19 inpatients | 60 | Treatment group: Mean age 51, 50% male  Control group: Mean age 53, 53.3% male | Vitamin A; Vitamin C; Vitamin E | To evaluate whether multivitamins can reduce the inflammatory markers, mortality rate, and duration of hospitalization in ICU-admitted patients with COVID-19 | Significant improvements in inflammatory markers and prolonged hospitalization rate in intervention group. |
| Ben Abdallah 2023[43] | Tunisia | Positive COVID-19 inpatients and outpatients | 470 | Treatment group:  Mean age 54.6, 52.4% male  Control group:  Mean age: 53.7, 53.6% male | Zinc | Evaluate the effect of zinc  supplementation in non-critically ill patients with COVID-19 | Oral zinc treatment for 15 days is associated with ~40% in mortality and ICU admission, with shortening symptom duration. |
| Coppock 2022[38] | United States | Positive COVID-19 inpatients | 66 | Treatment group: Mean age 61, 50% male, 63.6% Caucasian/white, 18.2% African American  Control group: Mean age 60 years, 50% male, 52.3% Caucasian/white, 38.6% African American | Vitamin C | Evaluate clinical improvement within 72 hours of pharmacological intravenous high-dose ascorbic acid treatment in patients with acute COVID-19 | Clinical improvement was not met in the study; vitamin C may improve supplemental oxygen use and reduce the time to patient discharge. |
| Darban 2021[21] | Iran | Positive COVID-19 inpatients | 20 | Mean age 59 +/- 19, 65% male | Vitamin C; Zinc | Assess the effect of combined intravenous vitamin C, oral melatonin, and oral zinc on improving clinical outcomes of hospitalized patients with severe COVID-19 | Intervention group did not have considerable improvement in clinical symptoms compared to placebo. |
| Fogleman 2022[37] | United States | Positive COVID-19 outpatients | 104 | Treatment group: Median age 50, 41% male  Control group: Median age 54, 29% male | Vitamin C | This study aimed to help determine the effect of dietary supplements on symptom course and quality of life in patients with mild-to-moderate COVID-19 infection. | Symptom score improved in vitamin C group vs. placebo group. |
| Hakamifard 2022[32] | Iran | Positive COVID-19 inpatients | 72 | Treatment group: Mean age 25.65, 63.2% male  Control group: Mean age 37.1, 64.7% male | Vitamin C; Vitamin E | Evaluate the effects of oral vitamin C and vitamin E as adjunctive therapy with the standard treatment in the outcome of COVID-19 patients | Co-administration of vitamin E and vitamin C did not significantly improve hospitalization time, and clinical symptoms. |
| Hellou 2022[40] | Israel | Positive COVID-19 inpatients | 50 | Treatment group: Mean age 52 +/- 14, 17% male  Control group: Mean age 53 +/- 14, 8% male | Vitamin C | Examine the safety and tolerability of ArtemiC oral spray in hospitalized COVID-19 patients and its efficacy in improving major symptoms of these patients. | Intervention group showed significantly greater clinical improvement (NEWS2 score) compared to placebo. |
| JamaliMoghadamSiahkali 2021[22] | Iran | Positive COVID-19 inpatients | 60 | Treatment group:  Mean age 57.53, 50% male  Control group: Mean age 61, 50% male | Vitamin C | Assess the efficacy of adding high-dose intravenous vitamin C (HDIVC) to the regimens for patients with severe COVID-19 disease | Peripheral oxygen saturation and body temperature improved in placebo and vitamin C groups, |
| Kumar 2022[41] | India | Positive COVID-19 outpatients | 260 | Treatment group: Mean age 37.4 +/- 10.4, 55% male  Control group: Mean age 36 +/-12, 56% male | Vitamin C; Vitamin E; Zinc; Selenium | Assess the effectiveness of the APMV2020 combination in reducing symptoms that is more subjects getting relieved of symptoms, reducing inflammatory markers (CRP, LDH, ferritin) and faster clinical recovery in mild and moderate COVID-19 patients. | Clinical parameters such as recovery time, and inflammatory markers were reduced in the intervention group. |
| Kumari 2020[19] | Pakistan | Positive COVID-19 outpatients | 150 | 56.9% male  Treatment group: 52 +/- 11  Control group: 53 +/-12 | Vitamin C | To find out the role of Vitamin C (VC) as adjunctive therapy in COVID-19 | Vitamin C can significantly improve clinical symptoms and reduce days of hospitalization, but not on mortality and mechanical ventilation requirements. |
| Labbani-Motlagh 2022[33] | Iran | Positive COVID-19 inpatients | 74 | Treatment group: Mean age 57.84 +/- 14.72, 59.5%  Control group: Mean age 58.89 +/- 14.4, 54.1% male | Vitamin C | To evaluate the protective potentials of high-dose  Vitamin C in the progression of coronavirus disease 2019 (COVID-19). | Vitamin C did not improve any clinical or biological parameters and could not reduce mortality rate at day 28. |
| Mahjoub 2023[42] | Tunisia | Positive COVID-19 outpatients | 164 | Treatment group: Mean age 35.6 +/- 8.4, 39% male  Control group: Mean age 34.3 +/- 9.9, 42.7% male | Vitamin A; Vitamin C; Vitamin E; Zinc | Examine the efficacy and safety of the association of melatonin, zinc and multivitamin supplements (containing vitamins A, C and E) in the treatment of COVID-19 and COVID-like illnesses | Intervention group had significant reduction in symptom duration. |
| Majeed 2021 [7] | India | Positive COVID-19 inpatients | 92 | Treatment group: Mean age 39.04 ± 7.70  Control group: Mean age 37.28 ± 7.40 | Zinc, Selenium | Examine the efficacy and safety of ImmuActive (Zinc, Selenium) as an adjunct therapy for COVID-19 patients | Intervention may reduce the severity of COVID-19 in patients. |
| Majidi 2021[23] | Iran | Positive COVID-19 inpatients | 100 | Treatment group: Mean age 59.2, 61% male  Control group: Mean age 63.82, 58% male | Vitamin C | To investigate the effect of vitamin C supplementation on pathological parameters and survival duration of critically ill patients with COVID-19. | Vitamin C supplementation increased survival duration. |
| Patel 2021[29] | Australia | Positive COVID 19 inpatients | 33 | Treatment group: Mean age 59.8 +/- 16.8, 73.3% male  Control group: Mean age 63.8 +/- 16.9, 55.5% male | Zinc | To evaluate the safety, feasibility, and biological effect of administering high‐dose intravenous zinc to COVID‐19 patients | Zinc assisted in improving mineral deficiency in COVID-19 patients in both groups. |
| Reino-Gelardo 2023[30] | Spain | Positive COVID-19 inpatients | 139 | Treatment group: Mean age 70, 57.1% male  Control group: Mean age 69, 55.1% male | Zinc; Selenium | To evaluate the potential effect of a food supplement (probiotics, prebiotics, vitamin D, zinc and selenium) in patients admitted with COVID-19. | Intervention group had significantly reduced duration of gastrointestinal symptoms. Intervention group had more patients in recovery. |
| Ried 2021[31] | Turkey | Positive COVID-19 inpatients | 237 | Mean age 63.3 +/- 15.7, 50% male | Vitamin C; Zinc | To assess the effectiveness of HCQ, AZM, and zinc with or without IVC in hospitalized patients with COVID-19 in reducing symptom severity and duration and preventing mortality | IV vitamin C contributes to a faster recovery in combination with zinc and standard treatment. |
| Rohani 2022[34] | Iran | Positive COVID-19 outpatients | 182 | Treatment group: 59.5% male  Control group: 56% male | Vitamin A | Determining the role of vitamin A supplement therapy for improving treatment outcomes in patients with COVID-19 | Vitamin A is efficacious in improving some clinical and paraclinical symptoms. |
| Somi 2022[35] | Iran | Positive COVID-19 inpatients | 30 | Mean age 60.21 +/- 13.61, 63.3% male | Vitamin A | To assess the effect of vitamin A supplementation on Coronavirus disease-2019 (COVID-19) in hospitalized patient. | No significant differences between vitamin A and placebo groups in terms of ICU admission rate, respiratory support and time to clinical response. |
| Tehrani 2022[36] | Iran | Positive COVID-19 inpatients | 54 | Treatment group: Mean age 61 +/- 17, 69% male  Control group: Mean age 58+/- 19, 44% male | Vitamin C | Assess the effectiveness of high dose intravenous vitamin C in patients with COVID-19 pneumonia | Improvement in peripheral oxygen saturation and respiratory rate in pneumonia patients with COVID-19 treated with vitamin C. |
| Thomas 2021[24] | United States | Positive COVID-19 outpatients | 214 | Mean age 45.12 +/- 14.6, 38.3% male | Vitamin C; Zinc | To examine whether high-dose zinc and/or high-dose ascorbic acid reduce the severity or duration of symptoms compared with usual care among ambulatory patients with SARS-CoV-2 infection. | Intervention groups did not significantly shorten the duration of symptoms compared to standard care. |
| Yang 2022[39] | China | Positive COVID-19 inpatients | 60 | Age range 46.1 +/- 9.2 – 50.3 +/- 9.5, 52% male | Vitamin C; Vitamin E | Determine whether traditional Chinese medicine alone (herbal decoction, oral vitamin E capsule, folic acid, vitamin C) or in combination with high dose intravenous vitamin C can improve therapeutic effect of Western medicine on COVID-19 | Clinical symptoms such as recovery time, symptom disappearance, and inflammatory markers were improved for the intervention group receiving antioxidants. |
| Zhang 2021[25] | China | Positive COVID-19 inpatients | 56 | Treatment group: Mean age 66.3 +/- 11.2, 55.6% male  Control group: Mean age 67.0 +/- 14.3, 75.9% | Vitamin C | Assess the efficacy of high-dose intravenous vitamin C together with conventional therapy as treatment for adult patients admitted to the ICU due to severe COVID-19 | High dose vitamin C did not improve invasive mechanical ventilation-free days in 28 days but improves the P/F ratio. |
