# Supplementary material for: Role of Antioxidant Therapy in the Treatment and Prognosis of COVID-19: A Systematic Review and Meta-analysis of Randomized Controlled Trials
Source: Curr Dev Nutr. 2024 Mar 24;8(5):102145. doi: 10.1016/j.cdnut.2024.102145 (PMC11061685; doi:10.1016/j.cdnut.2024.102145)
Supplement: Multimedia component 2 [file mmc2.docx]

**Supplementary Methods 1** – Search Strategy

**Database: Ovid MEDLINE(R) and Epub Ahead of Print, In-Process, In-Data-Review & Other Non-Indexed Citations <1946 to July 05, 2023>**

**Search Strategy:**

--------------------------------------------------------------------------------

1 COVID-19/ (229186)

2 SARS-CoV-2/ (155477)

3 (coronavirus/ or betacoronavirus/ or coronavirus infections/) and (disease outbreaks/ or epidemics/ or pandemics/) (40229)

4 (nCoV* or 2019nCoV or 19nCoV or COVID19* or COVID or SARS-COV-2 or SARSCOV-2 or SARS-COV2 or SARSCOV2 or SARS coronavirus 2 or Severe Acute Respiratory Syndrome Coronavirus 2 or Severe Acute Respiratory Syndrome Corona Virus 2).ti,ab,kf,nm,ot,ox,rx,px. (357190)

5 ((new or novel or "19" or "2019" or Wuhan or Hubei or China or Chinese) adj3 (coronavirus* or corona virus* or betacoronavirus* or CoV or HCoV)).ti,ab,kf,ot. (91996)

6 ((coronavirus* or corona virus* or betacoronavirus*) adj3 (pandemic* or epidemic* or outbreak* or crisis)).ti,ab,kf,ot. (15803)

7 ((Wuhan or Hubei) adj5 pneumonia).ti,ab,kf,ot. (421)

8 or/1-7 [Adapted CADTH's Ovid Medline COVID-19 Search Filter https://covid.cadth.ca/literature-searching-tools/cadth-covid-19-search-strings/#covid-19-medline ] (370201)

9 (covid* not covidence).tw,kf. (329640)

10 8 or 9 [COVID-19 SEARCH STRING] (370828)

11 limit 10 to yr="2019 -Current" (368824)

12 Antioxidants/ (152198)

13 antioxidant*.tw,kf. (275834)

14 anti-oxidant*.tw,kf. (14281)

15 exp Vitamin A/ (47417)

16 vitamin a.tw,kf. (27464)

17 retinol.tw,kf. (15308)

18 retinoic acid.tw,kf. (35195)

19 exp Ascorbic Acid/ (45400)

20 Ascorbic Acid.tw,kf. (37940)

21 vitamin c.tw,kf. (27852)

22 exp Vitamin E/ (34700)

23 vitamin e.tw,kf. (31544)

24 tocopherol*.tw,kf. (23245)

25 Selenium/ (23913)

26 selenium.tw,kf. (34902)

27 exp Zinc/ (67257)

28 zinc.tw,kf. (142635)

29 Copper/ (77862)

30 copper.tw,kf. (127258)

31 exp Carotenoids/ (94437)

32 (carotene* or carotenoid* or tetraterpene*).tw,kf. (39970)

33 Lycopene/ (3086)

34 Lycopene.tw,kf. (6175)

35 or/12-34 [ANTIOXIDANTS] (797383)

36 11 and 35 (2897)

37 exp randomized controlled trial/ (596784)

38 controlled clinical trial.pt. (95346)

39 (randomized or randomised).ab. (725957)

40 placebo.ab. (239378)

41 drug therapy.fs. (2602249)

42 randomly.ab. (411254)

43 trial.ab. (653665)

44 groups.ab. (2535763)

45 or/37-44 [ Cochrane MEDLINE RCT search filter Sensitivity-maximizing version (2023 revision); randomised added] (5711994)

46 36 and 45 (718)

47 46 not (exp animals/ not humans.sh.) (691)

48 limit 47 to english language (682)

49 remove duplicates from 48 (681)

**EBM Reviews - Cochrane Central Register of Controlled Trials <June 2023>**

1 COVID-19/ 4440

2 SARS-CoV-2/ 2270

3 (coronavirus/ or betacoronavirus/ or coronavirus infections/) and (disease outbreaks/ or epidemics/ or pandemics/) 335

4 (nCoV* or 2019nCoV or 19nCoV or COVID19* or COVID or SARS-COV-2 or SARSCOV-2 or SARS-COV2 or SARSCOV2 or SARS coronavirus 2 or Severe Acute Respiratory Syndrome Coronavirus 2 or Severe Acute Respiratory Syndrome Corona Virus 2).ti,ab,kf,ot. 16742

5 ((new or novel or "19" or "2019" or Wuhan or Hubei or China or Chinese) adj3 (coronavirus* or corona virus* or betacoronavirus* or CoV or HCoV)).ti,ab,kf,ot. 3888

6 ((coronavirus* or corona virus* or betacoronavirus*) adj3 (pandemic* or epidemic* or outbreak* or crisis)).ti,ab,kf,ot. 357

7 ((Wuhan or Hubei) adj5 pneumonia).ti,ab,kf,ot. 31

8 or/1-7 [Adapted CADTH's Ovid Medline COVID-19 Search Filter https://covid.cadth.ca/literature-searching-tools/cadth-covid-19-search-strings/#covid-19-medline ] 17089

9 (covid* not covidence).tw,kf. 16287

10 8 or 9 [COVID-19 SEARCH STRING] 17353

11 limit 10 to yr="2019 -Current" 17161

12 Antioxidants/ 5377

13 antioxidant*.tw,kf. 13241

14 anti-oxidant*.tw,kf. 1020

15 exp Vitamin A/ 2323

16 vitamin a.tw,kf. 2564

17 retinol.tw,kf. 1665

18 retinoic acid.tw,kf. 1015

19 exp Ascorbic Acid/ 2551

20 Ascorbic Acid.tw,kf. 1892

21 vitamin c.tw,kf. 4075

22 exp Vitamin E/ 2838

23 vitamin e.tw,kf. 4386

24 tocopherol*.tw,kf. 2020

25 Selenium/ 854

26 selenium.tw,kf. 2181

27 exp Zinc/ 1861

28 zinc.tw,kf. 6170

29 Copper/ 574

30 copper.tw,kf. 2040

31 exp Carotenoids/ 4224

32 (carotene* or carotenoid* or tetraterpene*).tw,kf. 2711

33 Lycopene/ 295

34 Lycopene.tw,kf. 679

35 or/12-34 [ANTIOXIDANTS] 35535

36 11 and 35 402

37 limit 36 to english language 395

38 remove duplicates from 37 394

**Embase Classic+Embase <1947 to 2023 July 05>**

1 antioxidant/ 190050

2 antioxidant*.tw,kf. 352280

3 anti-oxidant*.tw,kf. 23376

4 exp retinol/ 53727

5 vitamin a.tw,kf. 34835

6 retinol.tw,kf. 18986

7 ascorbic acid/ 120206

8 Ascorbic Acid.tw,kf. 49814

9 vitamin c.tw,kf. 35163

10 exp tocopherol/ 90173

11 vitamin e.tw,kf. 40217

12 tocopherol*.tw,kf. 27886

13 selenium/ 48805

14 selenium.tw,kf. 43045

15 zinc/ 146179

16 zinc.tw,kf. 171425

17 copper/ 148050

18 copper.tw,kf. 147230

19 exp carotenoid/ 189689

20 (carotene* or carotenoid* or tetraterpene*).tw,kf. 44917

21 lycopene/ 8600

22 Lycopene.tw,kf. 7223

23 or/1-22 1084674

24 coronavirus disease 2019/ or asymptomatic coronavirus disease 2019/ or exp covid-19 skin manifestation/ or covid-19-associated acute hemorrhagic necrotizing encephalopathy/ or exp covid-19-associated coagulopathy/ or covid-19-associated meningoencephalitis/ or covid-19-associated nephropathy/ or pediatric multisystem inflammatory syndrome/ 369329

25 exp Severe acute respiratory syndrome coronavirus 2/ 103292

26 (coronavirinae/ or betacoronavirus/ or coronavirus infection/) and (epidemic/ or pandemic/) 9478

27 (nCoV* or 2019nCoV or 19nCoV or COVID19* or COVID or SARS-COV-2 or SARSCOV-2 or SARS-COV2 or SARSCOV2 or SARS coronavirus 2 or Severe Acute Respiratory Syndrome Coronavirus 2 or Severe Acute Respiratory Syndrome Corona Virus 2).ti,ab,kw,hw,ot. 441004

28 ((new or novel or "19" or "2019" or Wuhan or Hubei or China or Chinese) adj3 (coronavirus* or corona virus* or betacoronavirus* or CoV or HCoV)).ti,ab,kw,hw,ot. 387315

29 ((coronavirus* or corona virus* or betacoronavirus*) adj3 (pandemic* or epidemic* or outbreak* or crisis)).ti,ab,kw,ot. 20571

30 ((Wuhan or Hubei) adj5 pneumonia).ti,ab,kw,ot. 620

31 covid*.mp. not covidence.tw,kf. [mp=title, abstract, heading word, drug trade name, original title, device manufacturer, drug manufacturer, device trade name, keyword heading word, floating subheading word, candidate term word] 415311

32 or/24-31 [Adapated from CADTH's COVID-19 Search filter for Embase https://covid.cadth.ca/literature-searching-tools/cadth-covid-19-search-strings/ ] 484542

33 23 and 32 5713

34 exp "randomized controlled trial (topic)"/ 264097

35 exp controlled clinical trial/ 989765

36 randomization/ 99836

37 double blind procedure/ 214348

38 intermethod comparison/ 298865

39 (randomized or randomised or placebo or randomly).tw,kf. or trial.ab. or groups.ab. 5182223

40 34 or 35 or 36 or 37 or 38 or 39 5732402

41 33 and 40 983

42 41 not ((exp animal/ or animal experiment/ or nonhuman/) not (exp human/ or human experiment/)) 926

43 limit 42 to (english language and yr="2019 -Current") 898

44 limit 43 to embase 727

45 remove duplicates from 44 726

**International Pharmaceutical Abstracts <1970 to June 2023>**

1 (nCoV* or 2019nCoV or 19nCoV or COVID19* or COVID or SARS-COV-2 or SARSCOV-2 or SARS-COV2 or SARSCOV2 or SARS coronavirus 2 or Severe Acute Respiratory Syndrome Coronavirus 2 or Severe Acute Respiratory Syndrome Corona Virus 2).mp. [mp=title, subject heading word, registry word, abstract, trade name/generic name] 3790

2 ((new or novel or "19" or "2019" or Wuhan or Hubei or China or Chinese) adj3 (coronavirus* or corona virus* or betacoronavirus* or CoV or HCoV)).mp. [mp=title, subject heading word, registry word, abstract, trade name/generic name] 1325

3 ((coronavirus* or corona virus* or betacoronavirus*) adj3 (pandemic* or epidemic* or outbreak* or crisis)).mp. [mp=title, subject heading word, registry word, abstract, trade name/generic name] 153

4 ((Wuhan or Hubei) adj5 pneumonia).mp. [mp=title, subject heading word, registry word, abstract, trade name/generic name] 4

5 (covid* not covidence).mp. [mp=title, subject heading word, registry word, abstract, trade name/generic name] 4223

6 1 or 2 or 3 or 4 or 5 4764

7 (antioxidant* or anti-oxidant* or vitamin a or retinol or retinoic acid or Ascorbic Acid or vitamin c or vitamin e or tocopherol* or selenium or zinc or copper or carotene* or carotenoid* or tetraterpene* or Lycopene).mp. [mp=title, subject heading word, registry word, abstract, trade name/generic name] 23210

8 6 and 7 93

9 limit 8 to (english language and yr="2019 -Current") 75

10 (random* or RCT or double blind or trial*).mp. [mp=title, subject heading word, registry word, abstract, trade name/generic name] 96010

11 9 and 10 11

**Scopus**

1398 results

(( TITLE-ABS-KEY ( ( covid19* OR covid OR "SARS-COV-2" OR "SARSCOV-2" OR "SARS-COV2" OR sarscov2 OR ncov* OR 2019ncov OR 19ncov ) ) ) AND ( TITLE-ABS-KEY ( ( antioxidant* OR "anti oxidant*" OR "vitamin a" OR retinol OR "retinoic acid" OR "Ascorbic Acid" OR "vitamin c" OR "vitamin e" OR tocopherol* OR selenium OR zinc OR copper OR carotene* OR carotenoid* OR tetraterpene* OR lycopene ) ) )) AND ((TITLE-ABS-KEY((*"clinical trial*" OR "Controlled Trial*" OR rct OR random* OR "double blind*" or "single blind*" or placebo* OR "Cross over stud*" OR "Cross over trial *" OR "factorial design" or "control* group*"))) OR (INDEXTERMS ( "clinical trials" OR "clinical trials as a topic" OR "randomized controlled trial" OR "Randomized Controlled Trials as Topic" OR "controlled clinical trial" OR "Controlled Clinical Trials" OR "random allocation" OR "Double-Blind Method" OR "Single-Blind Method" OR "Cross-Over Studies" OR "Placebos" OR "multicenter study" OR "double blind procedure" OR "single blind procedure" OR "crossover procedure" OR "clinical trial" OR "controlled study" OR "randomization" OR "placebo" ))) AND NOT (TITLE ( animal* OR nonhuman* OR veterinar* OR avian* OR baboon* OR bird* OR bovine OR canine OR cat OR cats OR cattle* OR chick* OR chimp* OR cow OR cows OR dog OR dogs OR duck OR feline OR fish* OR geese OR goose OR macaque* OR marmoset* OR mice OR mouse OR murine OR ovine OR pig OR pigs OR piglet* OR porcine OR primate* OR rabbit OR rat OR rats OR rodent* OR sheep OR swine OR trout* OR zebrafish* ) AND NOT ( human* OR patient* OR women OR woman OR men OR man )) AND ( EXCLUDE ( DOCTYPE,"re" ) OR EXCLUDE ( DOCTYPE,"le" ) OR EXCLUDE ( DOCTYPE,"ed" ) OR EXCLUDE ( DOCTYPE,"cp" ) OR EXCLUDE ( DOCTYPE,"ch" ) OR EXCLUDE ( DOCTYPE,"cr" ) OR EXCLUDE ( DOCTYPE,"bk" ) ) AND ( LIMIT-TO ( LANGUAGE,"English" ) )

**Search History**

**Interface - EBSCOhost Research Databases**

**Search Screen - Advanced Search**

**Database - CINAHL Complete**

| \| **#** \| **Query** \| **Limiters/Expanders** \| **Results** \| \| --- \| --- \| --- \| --- \| \| S19 \| S17 AND S18 \| Limiters - English Language Expanders - Apply equivalent subjects Search modes - Boolean/Phrase \| 96 \| \| S18 \| (randomized controlled trials OR MH double-blind studies OR MH single-blind studies OR MH random assignment OR MH pretest-posttest design OR MH cluster sample OR TI (randomised OR randomized) OR AB (random*) OR TI (trial) OR (MH (sample size) AND AB (assigned OR allocated OR control)) OR MH (placebos) OR PT (randomized controlled trial) OR AB (control W5 group) OR MH (crossover design) OR MH (comparative studies) OR AB (cluster W3 RCT)) NOT ((MH animals+ OR MH animal studies OR TI animal model*) NOT MH human) \| Expanders - Apply equivalent subjects Search modes - Boolean/Phrase \| 968,957 \| \| S17 \| S7 AND S16 \| Expanders - Apply equivalent subjects Search modes - Boolean/Phrase \| 569 \| \| S16 \| S8 OR S9 OR S10 OR S11 OR S12 OR S13 OR S14 OR S15 \| Expanders - Apply equivalent subjects Search modes - Boolean/Phrase \| 74,963 \| \| S15 \| (antioxidant* OR "anti oxidant*" OR "vitamin a" OR retinol OR "retinoic acid" OR "Ascorbic Acid" OR "vitamin c" OR "vitamin e" OR tocopherol* OR selenium OR zinc OR copper OR carotene* OR carotenoid* OR tetraterpene* OR lycopene ) \| Expanders - Apply equivalent subjects Search modes - Boolean/Phrase \| 73,291 \| \| S14 \| (MH "Lycopene") \| Expanders - Apply equivalent subjects Search modes - Boolean/Phrase \| 668 \| \| S13 \| (MH "Carotenoids+") \| Expanders - Apply equivalent subjects Search modes - Boolean/Phrase \| 11,099 \| \| S12 \| (MH "Copper") \| Expanders - Apply equivalent subjects Search modes - Boolean/Phrase \| 2,538 \| \| S11 \| (MH "Zinc") OR (MH "Zinc Compounds+") \| Expanders - Apply equivalent subjects Search modes - Boolean/Phrase \| 6,537 \| \| S10 \| (MH "Selenium") OR (MH "Selenium Compounds") \| Expanders - Apply equivalent subjects Search modes - Boolean/Phrase \| 2,842 \| \| S9 \| (MH "Vitamin A") OR (MH "Beta Carotene") OR (MH "Ascorbic Acid") OR (MH "Vitamin E") \| Expanders - Apply equivalent subjects Search modes - Boolean/Phrase \| 14,124 \| \| S8 \| (MH "Antioxidants") \| Expanders - Apply equivalent subjects Search modes - Boolean/Phrase \| 22,785 \| \| S7 \| S1 OR S2 OR S3 OR S4 OR S5 OR S6 \| Limiters - Published Date: 20190101-20241231 Expanders - Apply equivalent subjects Search modes - Boolean/Phrase \| 142,072 \| \| S6 \| TI coronavirus \| Expanders - Apply equivalent subjects Search modes - Boolean/Phrase \| 9,199 \| \| S5 \| ((coronavirus* or corona virus* or betacoronavirus*) N3 (pandemic* or epidemic* or outbreak* or crisis)) \| Expanders - Apply equivalent subjects Search modes - Boolean/Phrase \| 10,507 \| \| S4 \| ((new or novel or "19" or "2019" or Wuhan or Hubei or China or Chinese) N3 (coronavirus* or corona virus* or betacoronavirus* or CoV or HCoV)) \| Expanders - Apply equivalent subjects Search modes - Boolean/Phrase \| 19,151 \| \| S3 \| nCoV* or 2019nCoV or 19nCoV or COVID* or SARS-COV-2 or SARSCOV-2 or SARS-COV2 or SARSCOV2 or SARS coronavirus 2 or Severe Acute Respiratory Syndrome Coronavirus 2 or Severe Acute Respiratory Syndrome Corona Virus 2 \| Expanders - Apply equivalent subjects Search modes - Boolean/Phrase \| 140,089 \| \| S2 \| (MH "SARS-CoV-2") \| Expanders - Apply equivalent subjects Search modes - Boolean/Phrase \| 1,385 \| \| S1 \| (MH "COVID-19") \| Expanders - Apply equivalent subjects Search modes - Boolean/Phrase \| 41,987 \| |
| --- | --- | --- | --- | --- | --- | --- | --- | --- | --- | --- | --- | --- | --- | --- | --- | --- | --- | --- | --- | --- | --- | --- | --- | --- | --- | --- | --- | --- | --- | --- | --- | --- | --- | --- | --- | --- | --- | --- | --- | --- | --- | --- | --- | --- | --- | --- | --- | --- | --- | --- | --- | --- | --- | --- | --- | --- | --- | --- | --- | --- | --- | --- | --- | --- | --- | --- | --- | --- | --- | --- | --- | --- | --- | --- | --- | --- | --- | --- | --- | --- |
