# Supplementary material for: Role of Antioxidant Therapy in the Treatment and Prognosis of COVID-19: A Systematic Review and Meta-analysis of Randomized Controlled Trials
Source: Curr Dev Nutr. 2024 Mar 24;8(5):102145. doi: 10.1016/j.cdnut.2024.102145 (PMC11061685; doi:10.1016/j.cdnut.2024.102145)
Supplement: Multimedia component 3 [file mmc3.docx]

| Study ID (author, year) | Title | Journal | Antioxidant Type | Intended Purpose (Related to COVID) | Combo Treatment? | Study Design | Research question(s) | Results (linked to research question) | Jurisdiction | Unit(s) of Analysis | Number | Demographics | Is potential for bias identified/described? | If yes, which? (Gender, Sex, Ethnicity, SES) | Is potential for bias mitigated? If yes, how? | Target Population | Impact (as reported by author) |
| --- | --- | --- | --- | --- | --- | --- | --- | --- | --- | --- | --- | --- | --- | --- | --- | --- | --- |
|  |  |  |  |  |  |  |  |  |  |  |  |  |  |  |  |  |  |

**Supplementary Methods 2 – Data extraction forms**

**Data extraction form for systematic reviews:**

**Data extraction form for meta-analyses:**

| **Study ID (author, year)** | **Title** | **Treatment (type, dose, method of delivery)** | **Control arm** | **Outcomes of Interest (ex. ventilation, CRP)** | **Effect Measure (i.e., mean, OR, HR)** | **Baseline** | **Change/Final (T = treatment, C = control)** |
| --- | --- | --- | --- | --- | --- | --- | --- |
|  |  |  |  |  |  |  |  |
